# *Mitracarpus frigidus* in the Treatment of Vulvovaginal Candidiasis: A Comprehensive Evaluation of Its Therapeutic Properties

**DOI:** 10.1021/acsomega.5c05713

**Published:** 2025-10-17

**Authors:** Thalita F. Souza, Matheus T. Branca, Mariane R.C. Comitre, Débora A. de Oliveira, Lara M. Campos, Ari S. O. Lemos, Priscila L. Paula, Thayná G. Ferreira, Lívia R. Gamarano, Irley Olívia M. Diniz, Thiago P. Silva, Ana Bárbara Polo, Paula R. B. Dib, Eugênio D. Hottz, Nícolas Glanzmann, Ana Carolina M. Apolônio, Luciana M. Chedier, Renato Dantas-Medeiros, Marcelo G.F. Araújo, Rossana C. N. Melo, Rodrigo L. Fabri

**Affiliations:** † Bioactive Natural Products Laboratory, Department of Biochemistry, Institute of Biological Sciences, Federal University of Juiz de Fora, Juiz de Fora Campus, Juiz de Fora, Minas Gerais 36036-900, Brazil; ‡ Laboratory of Cellular Biology, Department of Biology, Institute of Biological Sciences, Federal University of Juiz de Fora, Juiz de Fora Campus, Juiz de Fora, Minas Gerais 36036-900, Brazil; § Laboratory of Bacterial Physiology and Molecular Genetics, Institute of Biological Sciences, Department of Parasitology, Microbiology, and Immunology, Federal University of Juiz de Fora, Juiz de Fora, Minas Gerais 36036-900, Brazil; ∥ Laboratory of Immunothrombosis, Department of Biochemistry, Institute of Biological Sciences, Federal University of Juiz de Fora, Juiz de Fora, Minas Gerais 36036-900, Brazil; ⊥ Department of Chemistry, Institute of Exact Sciences, Federal University of Juiz de Fora, Juiz de Fora, Minas Gerais 36036-900, Brazil; # Plant Chemistry Laboratory, Department of Botany, Institute of Biological Sciences, Federal University of Juiz de Fora, Juiz de Fora, Minas Gerais 36036-900, Brazil; ∇ Laboratory of Pharmacognosy, Department of Pharmaceutical Sciences, Faculty of Pharmacy, Federal University of Rio Grande Do Norte, Natal, Rio Grande Do Norte 59078-900, Brazil; ○ Laboratory of Pharmacology, Federal University of São João Del-Rei, Campus Centro Oeste Dona Lindu, Divinópolis, Minas Gerais 35501-296, Brazil

## Abstract

*Mitracarpus frigidus* (Rubiaceae) is a Brazilian native species traditionally used in folk medicine and has been associated with a range of biological activities, including anti-inflammatory, antibacterial, and antifungal effects. Among the relevant targets, *Candida* spp. stand out as opportunistic fungi responsible for infections such as vulvovaginal candidiasis, a widespread public health concern aggravated by the growing limitations and reduced efficacy of conventional antifungal therapies. Thus, this study investigated the cytotoxic and in vitro and in vivo antifungal activities of *M. frigidus* hexanic extract (MFH) against *Candida* species and its possible mechanism of action. Eleven compounds were identified in MFH, including pentalongin and stigmasterol. *In vitro* assays revealed that MFH exhibited minimum inhibitory concentrations (MIC) between 250 and 1000 μg/mL against *Candida* strains, indicating fungistatic effects. Mechanism of action assays revealed that MFH disrupted fungal cell envelopes, damaged mitochondrial membranes, and inhibited growth phases. MFH reduced fungal cell viability without causing mammalian cell cytotoxicity. In vivo, MFH reduced fungal infection in an experimental model of vulvovaginal candidiasis. These results emphasize the potential of *M. frigidus* as a therapeutic agent against *Candida*-associated infections, particularly vulvovaginal candidiasis, and encourage further studies, including those involving human volunteers.

## Graphical abstract

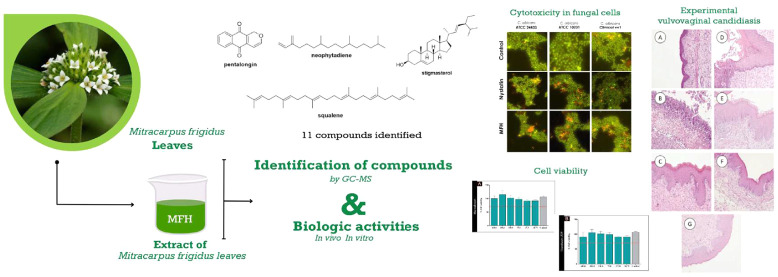

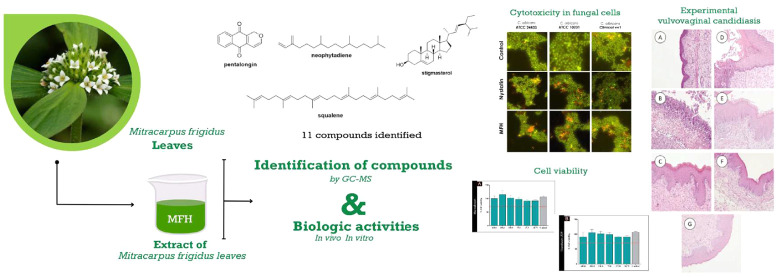

## 1 Introduction

*Candida* species are clinically relevant pathogens and represent a leading cause of systemic fungal infections, particularly in immunocompromised individuals and patients undergoing prolonged intensive care.^1,2^ One of the most frequent clinical manifestations is vulvovaginal candidiasis (VVC), which arises from colonization of the vaginal mucosa.^3^ The main etiological agents include *Candida albicans*, *Candida. tropicalis*, *Candida glabrata*, *Candida krusei*, *Candida parapsilosis*, and, more recently, the emerging multidrug-resistant species *Candida auris*.^4,5^

Most cases of VVC are attributed to *C. albicans*, a species capable of adhering to vaginal epithelial cells and triggering tissue damage accompanied by an inflammatory response. The condition is typically characterized by vaginal discharge and intense itching, which represent its main clinical symptoms. Besides, *C. albicans* infections are the leading cause of invasive infections, corresponding to mortality rates of 40%, even with current treatments.^6^ This species also raises concern over the development of resistance through multiple mechanisms, like changing the pharmacological target and expression of efflux pumps.^7^ Thus, there is a considerable demand for novel antifungals since a new class has not been into clinical practice since the 2000s and the prevalence of resistant strains of fungi is on the rise.^1,8^

*Mitracarpus frigidus* (Willd. ex Roem. and Schult.) K. Schum. (Rubiaceae) is a perennial herbaceous plant that is widespread in tropical America and the West Indies. In Brazil, t is found from the Amazon region in the north to the Rio Grande do Sul region in the south.^9^ This species, especially the methanolic extract of the aerial parts, exhibited several biological activities reported in the literature, such as antitumor,^10,11^ antioxidant, anti-inflammatory,^10,12^ schistosomicidal,^13^ and antimicrobial.^14–17^ Moreover, some compounds have already been identified in this plant species, such as kaempferol, rutin,^18^ kaempferol-*O*-rutenoside,^19^ ursolic acid, psychorrubrin,^10,13^ methyl ursolate,^12^ and scopoletin.^20^

Studies on lipophilic compounds from *M. frigidus* are scarce in the literature, mainly in the treatment of fungal infections. However, several lipophilic classes, such as fatty acids, terpenoids, and quinones have reported antifungal activities.^21–23^

Considering the importance of *M. frigidus* as a source of bioactive molecules, and the need for innovative alternative treatments for infections caused by multidrug-resistant *Candida* spp, in addition to the scarcity of scientific investigations on this species, this study aimed to investigate the cytotoxicity, in vitro and in vivo antifungal activities of the hexanic extract from *M. frigidus* aerial parts and its possible mechanism of action.

## 2 Material and Methods

### 2.1 Vegetal Material and Hexane Extract Preparation

Aerial parts of a small population with individuals of *M. frigidus* (Willd. Ex Roem Schult.) K. Schum were collected in Juiz de Fora, Minas Gerais, Brazil, at 08:00 a.m. in May 2021 (autumn), at the following coordinates 21°78′0,73S, 43°38′2,23W. Dr. Vinícius Antônio de Oliveira Dittrich from the Federal University of Juiz de Fora Botany Department identified the plant and the voucher was put on deposit at the Leopoldo Krieger Herbarium, CESJ 46076. In addition, the research group has the authorization to use the species under study SISGEN/Brazil A032F41. The plant name was confirmed on www.theplantlist.org (accessed May 06, 2024).

The hexane extract (MFH) was prepared according to Fabri et al.^24^ One hundred grams of dried and pulverized plant material was macerated statically with hexane. After extraction and filtration (3 × 500 mL), the solvent was then evaporated at 45 °C under reduced pressure using a rotary evaporator (Heidolph – Laborota 4000). After this step, the extract was weighed and its yield was calculated (22%). MFH was kept refrigerated until the biological and chemical tests were performed.

### 2.2 Chemical Characterization of MFH by Gas Chromatography Coupled to Mass Spectrometry (GC-MS)

Five milligrams of MFH were analyzed using a Hewlett-Packard 6890 gas chromatograph, coupled to an HP5972 mass spectrometer, equipped with a Wiley 7n.1 database. The capillary column used was the HP5MS type with 0.25 μm film thickness, 30 m length, and 250 μm inner diameter. Helium was employed as carrier gas (1 mL/min) and separations were carried out using a 250 °C injector and a 280 °C detector. The temperature was programmed to start at 70 °C and increase by 2 °C per minute until 290 °C. The operating parameters of the mass spectrometer were 70 eV, ion source, 250 °C, and electron impact. The fragmentation profile of the WILEY database and data from the literature were used to compare how similar the chemical components were. The analysis was performed in collaboration with Oswaldo Cruz Foundation (FIOCRUZ) through the Analytical Methods Platform of Farmanguinhos/FIOCRUZ.

### 2.3 Fourier Transform Infrared Spectroscopy

Fourier transform infrared (FTIR) spectra were obtained in an ALPHA FT-IR Bruker spectrometer in the 4000–400 cm^–1^ region. The spectra were acquired utilizing an accessory for attenuated total reflectance (Platinum ATR) integrated with the Bruker Alpha FTIR spectrometer, operating at a resolution of 2 cm^–1^ and accumulating 64 scans. Spectrum acquisition was conducted using the Bruker Opus software package version 6.0. Subsequently, the data were exported and processed using Origin 9.0.

### 2.4 *In Vitro* Antifungal Activity

#### 2.4.1 Fungal Strains

The following fungi were employed to test MFH antifungal effectiveness: *C. albicans* ATCC 24433, sensitive to conventional antifungal drugs; *C. albicans* ATCC 10231, resistant to anidulafungin, voriconazole, itraconazole, and fluconazole; *C. glabrata* ATCC 2001, sensitive to conventional antifungals; *C. krusei* ATCC 6258, intrinsically resistant to fluconazole; three clinical strains of *C. albicans* isolated from the vulvovaginal region (*C. albicans* vv1, *C. albicans* vv2 and *C. albicans* vv3); and two strains isolated from oral mucosa (*C. albicans* mo2 and *C. albicans* mo4).

#### 2.4.2 Determination of the Minimum Inhibitory Concentration of MFH

The assay was conducted under the procedure described by the Clinical and Laboratory Standards Institute (CLSI) to determine the Minimum Inhibitory Concentration (MIC).^25,26^ Fungal strains were previously cultured at 35 °C for 24 h in Sabouraud agar. MFH was prepared at 2.5 mg/mL in DMSO 1% and then diluted successively, obtaining concentrations from 1000 to 62.5 μg/mL in 96-well microplates, which already contained 100 μL of Roswell Park Memorial Institute broth 1640 (RPMI-1640). The inoculum was made from the dilution, at a ratio of 1:99, of the suspension containing 10^6^ CFU/mL, standardized using the 0.5 McFarland turbidity reference. Then, 20 μL of standardized fungal suspension were inoculated onto microplates to create a final volume of 200 μL, yielding a final concentration of 10^3^ CFU/mL in the well. The growth control underwent the same tests simultaneously (sterile water + RPMI-1640 broth + microorganism) and the blank of the experiment (RPMI-1640 broth + sterile water + extract). Nystatin (40 to 0.3 μg/mL) and fluconazole (10,000 to 78.1 μg/mL) were used as positive controls. Plates were incubated at 35 °C for 24 h. The experiments were performed in triplicate.

#### 2.4.3 Determination of the Minimum Fungicidal Concentration (MFC)

Aliquots of 10 μL were obtained from each well in the MIC assay that did not exhibit any fungal growth to determine the MFC and were grown on plates with Sabouraud agar. Then, the plates were incubated at 35 °C for 24 h.

After the MIC and MFC were determined, three strains of *C. albicans* were selected for the following assays. The selected strains were *C. albicans* ATCC 24433, *C. albicans* ATCC 10231, and a *C. albicans* clinical strain vv1.

#### 2.4.4 Fungal Killing Assay

This assay was performed to determine the effect of MFH on the microbial growth curve through the method described by Babii et al.^27^ with modifications. Previously grown fungal strains (10^6^ CFU/mL in sterile water) were inoculated in tubes containing RPMI-1640 broth and MFH at the MIC value, and incubated at 35 °C. The absorbance was measured at 600 nm at times 0, 4, 8, 12, 24, 30, 36, and 48 h of incubation. Nystatin (MIC value) was used as the positive control. As a growth control, fungus strains were inoculated in RPMI-1640 broth. The experiment was performed in triplicate. Two graphs were plotted based on the results: (i) absorbance (600 nm) × incubation time and (ii) area under the curve.

#### 2.4.5 Density of Fungal Cells

Fungal cell counts were performed on cytocentrifugation preparations.^28^ The chosen *C. albicans* strains were cultured at 35 °C for 24 h after being inoculated in tubes with RPMI-1640 broth containing MFH (MIC value). The positive controls were incubated with nystatin (MIC value) and the fungus strains were implanted in broth as growth controls. Thermo Electron’s Shandon cytospin 4 centrifuge was utilized to prepare the samples after fixing them in 4% formaldehyde and staining them with *Calcofluor White* (CW) at 5 μg/mL final concentration. Using immersion oil and a fluorescent microscope (Olympus BX-60), microorganisms on the slides were counted in 10 randomly selected fields at a magnification of 1000×. Equation 1 was used for quantification:

1 (n×a)(V×a)

where *n* = number of cells counted, *a* = area of the grid counted, and *V* = sample volume **×** volume counted/total volume.

### 2.5 Cell Envelope Analysis

#### 2.5.1 Crystal Violet Permeability Analysis

Crystal violet permeability assay was performed according to Devi et al.^29^ Freshly grown *C. albicans* strains were diluted in RPMI-1640 broth to 0.5 McFarland scale (10^6^ CFU/mL) and inoculated in tubes supplemented with MFH and nystatin (MIC values). Subsequently, the tubes were incubated at 35 °C for 4 h. Fungal cell suspensions were centrifuged at 1000 rpm for 10 min. The generated pellets were resuspended in a crystal violet solution at 10 μg/mL, and they were incubated once more for 10 min at 35 °C. The tubes were then centrifuged for 15 min at 1000 rpm, and a spectrophotometer was used to determine the optical density (OD) of the supernatant at 570 nm. The OD of the supernatant from the untreated cell group was used as the growth control, and the OD value of the crystal violet solution was considered 100%. All assays were carried out in triplicate. The percentage of crystal violet absorbance was determined using the following eq 2:

% violet crystal absorption=sample OD value⁢ OD/value of crystal
violet solution×100 2

#### 2.5.2 Membrane Leakage and Protein Content

The assay was performed according to the method described by Tang et al.^30^ The *C. albicans* strains were grown for 24 h at 35 °C in RPMI-1640 broth, after which they were centrifuged at 1000 rpm for 5 min, washed, and resuspended in 10 mM phosphate-buffered saline (PBS) solution (pH 7.4), where they attained a final density of about 10^6^ CFU/mL. The fungal strains were incubated with MFH and nystatin (MIC values) for different time intervals: 0, 1, 2, 3, and 4 h. PBS-incubated fungal cultures were employed as growth controls. After incubation, the cell suspensions were centrifuged at 3000 rpm for 15 min. A spectrophotometer was then used to measure the supernatants at 260 nm. The experiment was performed in triplicate.

For total protein dosage, the supernatants of the fungal strains cultured for 4 h were used after the measurements. This test was performed according to the method adapted from Lowry.^31^ Aliquots of 10 μL of the supernatants were transferred into test tubes containing water and 2 mL of the reactive solution (2% potassium sodium tartrate, 1% copper sulfate, and an alkaline mixture of NaOH and Na_2_CO_3_). Nystatin was included as a positive control. Subsequently, 2 mL of Folin reagent was added to each tube. The mixtures were homogenized and allowed to react for 30 min at room temperature. Absorbance readings were then taken at 660 nm using a spectrophotometer, and values were compared to a bovine serum albumin calibration curve. All assays were conducted in triplicate.

#### 2.5.3 Sorbitol Protection Assay

The sorbitol protection assay was performed according to Frost et al.^32^ after the MIC value for MFH was established. On a 96-well sterile microplate with RPMI-1640 culture medium enriched with 0.8 M sorbitol, a serial microdilution was carried out. The stock MFH solution was diluted to concentrations between 4000 and 31.25 μg/mL. The MIC values were calculated after the microplates had been incubated at 35 °C for 24 h. The experiment was carried out in triplicate.

#### 2.5.4 Exogenous Ergosterol Binding Assay

The exogenous ergosterol-binding assay was performed after determining the MIC value for MFH, as well as the sorbitol protection assay.^33^ On a 96-well sterile microplate, serial microdilution was carried out using RPMI-1640 culture media that was enhanced with ergosterol (400 μg/mL). The stock MFH solution was diluted to concentrations between 4000 and 31.25 μg/mL. As a positive control, nystatin was employed at concentrations ranging from 40 to 0.312 μg/mL. The microplate was then incubated for 24 h at 35 °C to ascertain the MIC values. The experiment was performed in triplicate.

#### 2.5.5 Determination of Ergosterol Content

*C. albicans* strains previously grown in sterile water (10^6^ CFU/mL) were aliquoted in previously weighed tubes and centrifuged at 1000 rpm for 5 min. One mL of RPMI-1640 broth, 500 μL of sterile water, and 500 μL of MFH and nystatin (MIC values) were added to each tube with the resulting pellet. Cells were incubated alone, with RPMI-1640 and sterile water, serving as growth control. After 24 h of incubation at 35 °C, the tubes were centrifuged at 1000 rpm for 5 min. The resulting *pellets* were washed with sterile water and weighed. Then, 1 mL of ethanol was added to each tube and left in an ultrasonic bath at 60 °C for 30 min. The tubes were centrifuged again at 1000 rpm for 5 min and the supernatants were read in a spectrophotometer at 282 and 230 nm. The experiment was performed in triplicate.^34^ The ergosterol content was calculated according to eq 3:

$$\mathrm{\% Ergosterol}=[(A_{281/290})\times F]/\mathrm{pellet weight}-\left[(A_{230/518})\times F\right]/\mathrm{pellet weight}\times 100$$ 3

Where A_281_ and A_230_ are the absorbance values read at 281 and 230 nm, respectively; F is the dilution factor; crystalline ergosterol and dehydroergosterol have values of 290 and 518, respectively.

#### 2.5.6 Mitochondrial Membrane Potential (MMP)

Tetramethylrhodamine (TMRE), a probe used to mark active mitochondria, was utilized to measure MMP.^35^ MFH or nystatin treatments (both at doses corresponding to the MIC values) were applied to the fungal cell suspensions (3 × 10^6^ CFU/mL) for 4 h. Standards such as oligomycin A (15 μg/mL) and 4-(trifluoromethoxy)­phenylhydrazone (FCCP, 10 μM), were utilized in cultures for 10 min before TMRE incubation, to control the experiment. As treated and untreated growth controls, cultures were incubated solely with buffers. Next, the cells were centrifuged for 5 min at 1500 rpm, then washed in HBSS solution and treated with 20 μM TMRE for 30 min at 35 °C. The sample was once again centrifuged and resuspended in HBSS buffer after labeling. A FACSCanto II flow cytometer (Becton and Dickinson) was used to quantitatively assess the average median fluorescence intensity (MFI) of the suspensions using 10.000 recorded events. The *FlowJo* program was utilized for the analyses, and the tests were performed in triplicate.

### 2.6 Cytotoxicity in Fungal Cells

Using the LIVE/DEAD *BacLight* kit (Molecular Probes), which contains two fluorescent markers (Syto 9 and propidium iodide (PI)); the intracellular metabolic activity of the fungi was measured to determine their viability. Selected *C. albicans* strains were prepared in sterile water (10^6^ CFU/mL) and then inoculated into tubes with RPMI-1640 broth containing MFH (MIC value). The positive control was nystatin. The tubes were incubated for 24 h at 35 °C. Fungal cells in suspension were stained with the reagents and then incubated for 30 min in the shelter of light. Samples were prepared for examination using cytocentrifugation at 452 g/10 min.^28^ The resulting slides were analyzed under a BX-60 fluorescence microscope (Olympus). Total cell count was performed in 10 random fields. Cell density was expressed by the total number of cells and the ratio of live/active and dead/inactive cells was established. The experiment was performed in triplicate.

### 2.7 Cytotoxicity in Mammalian Cells

#### 2.7.1 Culture of Peritoneal Macrophages and Fibroblast Cell Line L929 Strains

Macrophages were harvested from the peritoneal cavity of BALB/c mice previously injected with 3% thioglycolate medium and used for cell viability assays. Cells were seeded in microtiter plates at a density of 2 × 10^5^ cells per well in RPMI-1640 medium supplemented with 2 mM l-glutamine, 5% FBS, and 100 μg/mL penicillin/streptomycin, and incubated at 37 °C in a 5% CO_2_ atmosphere. All procedures involving animals were approved by the Animal Research Ethics Committee of the Federal University of Juiz de Fora (protocol no 007/2018-CEUA). Fibroblast L929 cells (ATCCCCL-1 NCTC) were cultured using the same conditions at a seeding density of 5 × 10^3^ cells per well.

#### 2.7.2 Evaluation of Cell Viability

The colorimetric assay using MTT (3-(4,5-dimethylthiazol-2-yl)-2,5-diphenyltetrazolium bromide) was used to determine cell viability after exposure to MFH.^36^ Cells were treated with varying concentrations of MFH (18.75–600 μg/mL) in triplicate, with the final volume adjusted to 100 μL per well. Plates were incubated for 48 h at 37 °C in a 5% CO_2_ atmosphere. After incubation, 10 μL of MTT solution (5 mg/mL) was added to each well, and the volume was brought up to 100 μL with RPMI-1640 medium. Cells were further incubated for 2 h and 30 min at 37 °C and 5% CO_2_ to allow formation of formazan crystals. Subsequently, the medium was removed, and 100 μL of DMSO was added to dissolve the crystals. Absorbance was measured at 595 nm after color stabilization. Dimethyl sulfoxide (0.06%) served as the negative control.

### 2.8 Experimental Vulvovaginal Candidiasis (VVC)

Female Wistar rats (150–200 g) were acclimated in the experimental room for at least 5 days prior to the start of the study. Animals were housed under controlled conditions (25 ± 1 °C, 12 h light/dark cycle) in the Laboratory of Pharmacology at UFSJ – Campus Centro-Oeste, following institutional guidelines. The experimental protocol was approved by the Animal Experimentation Ethics Committee of the Federal University of São João del-Rei (Protocol No. 021/2020). To establish a chronic and uniform infection, rats were immunosuppressed with a single intraperitoneal dose of cyclophosphamide (50 mg/kg b.w.) and estrus was induced by subcutaneous administration of estradiol cypionate (0.2 mg/mL) once daily for 4 consecutive days prior to infection. Animals were then inoculated intravaginally with 0.1 mL of *C. albicans* ATCC 10231 suspension (5 × 10^7^ CFU/mL) using a micropipette with disposable tips. Two days postinoculation (designated day 0), the vaginal fungal burden was assessed by collecting vaginal lavage with 0.1 mL of sterile saline and determining colony-forming units (CFU) on SDA plates. The infection was considered successfully established when the mean CFU from each rat reached at least 10^3^ CFU/mL.^37^

#### 2.8.1 Treatments in the Vulvovaginal Candidiasis (VVC) Model

To evaluate the effects of MFH on vulvovaginal candidiasis (VVC), infected Wistar female rats (*n* = 42) were randomly assigned to seven groups (*n* = 6 per group) and treated topically (0.1 mL) as follows: Group 1, noninfected control; Group 2, infected and untreated (negative control); Group 3, infected and treated with clotrimazole cream (CTZ, 10 mg/g, positive control); Group 4, infected and treated with MFH at 1.0 mg/mL; Group 5, infected and treated with MFH at 0.5 mg/mL; Group 6, infected and treated with MFH at 0.1 mg/mL; and Group 7, infected and treated with vehicle control (10% DMSO in distilled water). Treatments were applied once daily for six consecutive days. Vaginal fungal loads were assessed on days 3 and 6 postinfection to monitor treatment efficacy. On day 7, animals were euthanized, and vaginal and vulvar tissues were collected for histopathological evaluation. Samples were fixed in 10% buffered formalin, longitudinally trimmed, embedded in paraffin, and sectioned at 5 μm thickness. Sections were stained with Hematoxylin and Eosin (HE) to assess tissue responses to both *Candida* infection and the different treatment regimens.

### 2.9 Statistical Analysis

Statistical analysis of the trials was performed using GraphPad Prism 6.0 software. One-way ANOVA was used for the analysis of variance, followed by Bonferroni. At *p* < 0.05, differences were deemed significant. The results were expressed as mean ± standard deviation (SD).

## 3 Results and Discussion

### 3.1 Phytochemical Characterization of *Mitracarpus frigidus* Hexanic Extract

The phytochemical profile of *M. frigidus* hexanic extract (MFH) was initially characterized by GC-MS which indicated the presence of 12 signals in the chromatogram and 11 of them were identified as fatty acids (41.66%), terpenoids (16.16%), steroids (16.16%), alkaloids (8.33%), quinones (8.33%) and plastoquinone (8.33%), as shown in Table 1 and Figure 1. On the other hand, only one metabolite, belonging to the fatty acid lactone class with 6.5% of the chromatogram area was not identified (Supporting Information S1).

**Table 1:** Compounds Identified in *Mitracarpus frigidus* Hexanic Extract (MFH) by GC-MS

Compounds	Classes	RT (Min.)	Area %	Molecular weight (M+)	Main fragments	References
Methyl palmitate	Fatty acid methyl ester	33.33	7.63	270.20	270.20 ® 87.00 (70.01%); 74.00 (100.00%); 55.00 (28.53%); 43.00 (35.59%); 41.00 (27.61%)	Basumatary et al.^48^
Palmitic acid	Fatty acid	34.06	3.20	256.00	256.00 ® 185.00; 129.00; 73.00 (94.18%); 59.90 (72.85%); 55.00 (74.18%); 43.00 (75.58%); 41.00 (100.00%).	Abdel-Aal et al.;^49^ Pinto et al.^50^
2-Azaanthraquinone	Benzylisoquinoline alkaloid	34.64	5.64	209.00	209.00 (100.00%) ® 181.00 (34.23%); 153.0 (54.,06%); 126.00 (35.73%); 76.00; 49.90 (28.47%).	Gbaguidi et al.;^51^ Okunade et al.^52^
Methyl linoleate	Fatty acid methyl ester	36.54	3.56	294.10	294.10 ® 80.90 (66.56%); 79.00 (48.38%); 67.00 (100.00%); 55.00 (68.98%); 41.00 (72.39%).	Basumatary et al.^48^
Pentalongin	Pyranonaphthoquinone	36.65	19.62	212.00	212.00 (61.68%) ® 184.00 (91.28%); 156.00; 128.00 (100.00%); 79.00; 55.00 (61.40%); 41.00 (59.95%).	Pialat et al.^53^
Methyl oleate	Fatty acid methyl ester	36.75	7.45	296.00	296.00 ® 74.00 (52.25%); 69.00 (68.56%); 55.00 (100.00%); 43.00 (49.61%); 41.00 (64.87%).	Basumatary et al.^48^
Neophytadiene	Diterpene	36.85	13.93	278.30	278.30 ® 123.00 (67.03%); 95.10 (98.28%); 82.00 (86.36%); 68.00 (100.00%); 57.00; 55.00; 43.00; 41.00 (79.20%).	Abdel-Aal et al.;^49^ Pinto et al.;^50^ Abu-Shandia et al.^54^
Indeterminate	Fatty acid lactone	37.38	6.50	251.8	251.8 234.9; 222.1; 203.7; 153.9; 135.0; 123.0; 108.0; 95.0; 79.0 (100%); 67.0 (77.44%); 55.0 (67.88%); 43.0; 41.0 (41.07%)	-
Squalene	Triterpene	47.92	15.76	410.30	410.30 ® 341.20; 281.00; 123.00; 95.00 (19.39%); 81.00 (51.26%); 69.00 (100.00%); 67.00 (19.10%) 41.00 (31.15%).	Alagammal et al.^55^
Alpha-tocopherol	Plastoquinone	52.39	2.98	430.30	430.30 (100.00%) ® 165.10 (77.29%); 164.10 (23.98%).	Abu-Shandia et al.^54^
Stigmasterol	Sterol	54.88	7.26	412.20	412.20 (100) ® 351.30; 271.15 (57.54%); 255.20 (67.19%); 83.10 (61.19%); 55.10 (70.80%)	Pinto et al.^50^
γ- Sitosterol	Sterol	56.13	6.47	414.00	414.00 (100) ® 396.40 (49.76%); 381.00; 329.30 (54.92%); 303.30 (50.21%); 43.10 (45.70%).	Abdel-Aal et al.^49^
Total (%)			100	100		

**Figure 1:**
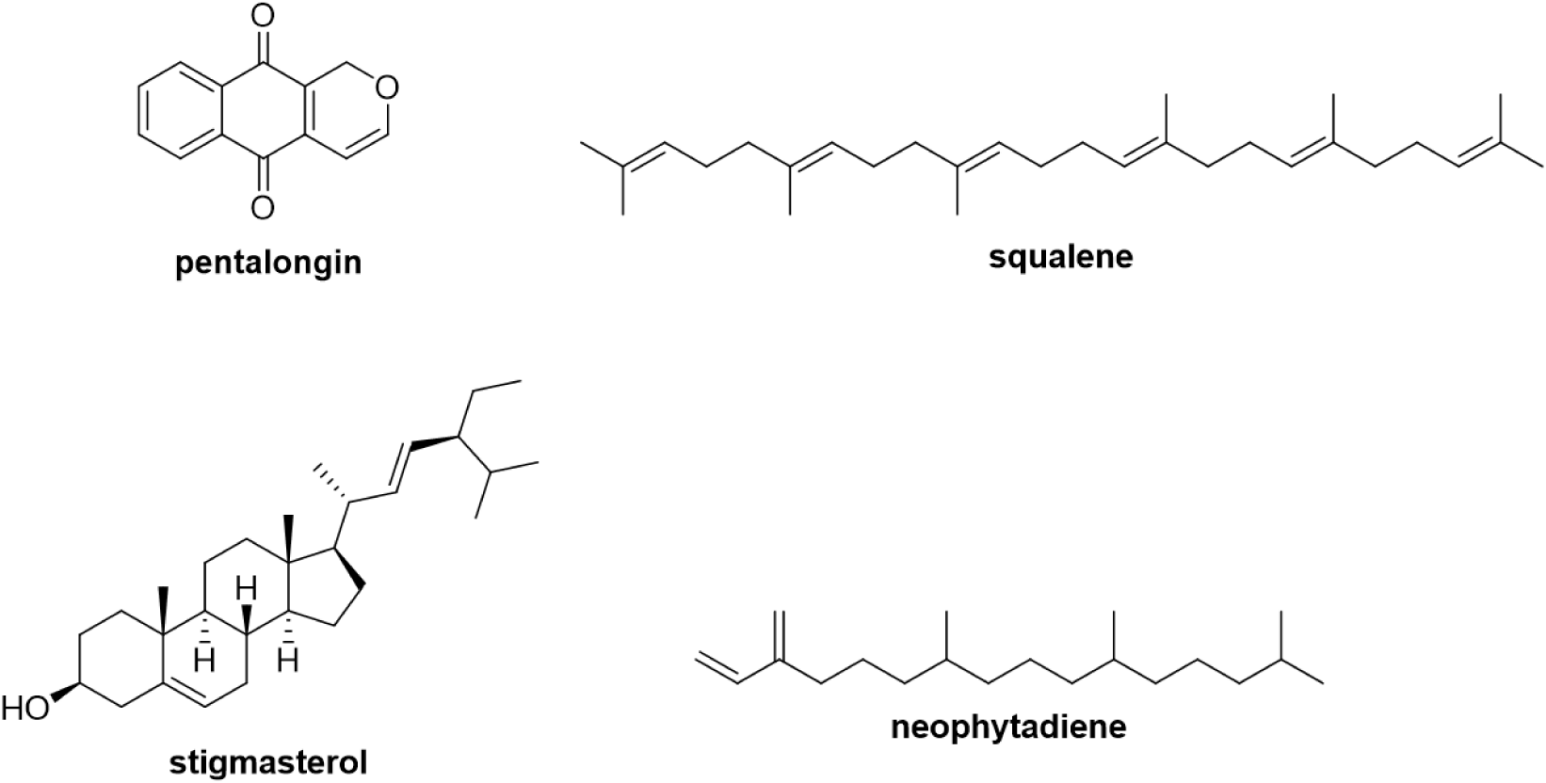
Main chemical constituents of *Mitracarpus frigidus* hexanic extract (MFH) identified by GC-MS.

According to GC-MS analysis, pentalongin was the major compound identified with 19.62% of the total area. This compound belongs to the class of pyranonaphthoquinones and has been found to possess antimicrobial, antiviral, anticancer, and antiparasitic properties.^38,39^ Squalene, the second most abundant compound with 15.76% area, is a hydrocarbon composed of six isoprene units. Squalene exhibits various beneficial properties, including anti-inflammatory, anticancer, antioxidant, and antimicrobial effects.^40,41^ Neophytadiene, a diterpene, was the third major compound identified; accounting for 13.93% of the chromatogram area and exhibiting previously reported antibacterial activity.^42^ Stigmasterol, γ-sitosterol, and 2-azaantraquinone were also identified from MFH. These compounds, except γ-sitosterol, have been previously isolated in species of the genus *Mitracarpus*, such as *Mitracarpus scaber* and *Mitracarpus villosus*,^43–45^ and have antioxidant, antibacterial, and antifungal activities.^28–47^ Thus, it is suggested that the antifungal activity of MFH against *C. albicans* may be related to synergistic interactions among these metabolites. Noteworthy, the presence of 2-azaantraquinone in other species of *Mitracarpus*, such as *M. villosus* and *M. scaber*, is evidence of a possible chemical mark of this genus.

The compounds were identified by comparing the chemical constituents with the fragmentation profile from the WILEY database and literature data. RT (Min) corresponds to the retention time in minutes and % area corresponds to the percentage area of the chromatogram.

Furthermore, the spectra obtained from the FTIR analysis of MFH (Figure 2), show prominent and broad peaks were observed at 2920 and 2850 cm^–1^, attributed to the stretching vibrations of C–H, supporting the chemical characterization by CG-MS indicating fatty acids as the major constituents.^56,57^ The peaks at 1733 and 1708 cm^–1^ correspond to characteristic carbonyl C=O stretching vibrations of ester and ketone bonds, respectively. These findings further support the presence of quinones.^58^ The C–H stretching vibrations of the terminal −CH_3_ methyl groups are notably weaker compared to those of the more abundant −CH_2_ groups, as indicated by the peaks at 1459 and 1377 cm^–1^, respectively. This observation strengthens the evidence for the presence of secondary metabolites categorized as hydrocarbons, including identified terpenes and sterols.^59^ Additionally, bands corresponding to carboxylic acids’ C–O stretching vibrations at 1243, 1170, 1089, 1027, and 1006 cm^–1^ were identified.^60^ Furthermore, the band observed at 719 cm^–1^, associated with the C–C stretching vibrations (aromatics), further supports the identification of alkaloids and other aromatic compounds.^60^

**Figure 2:**
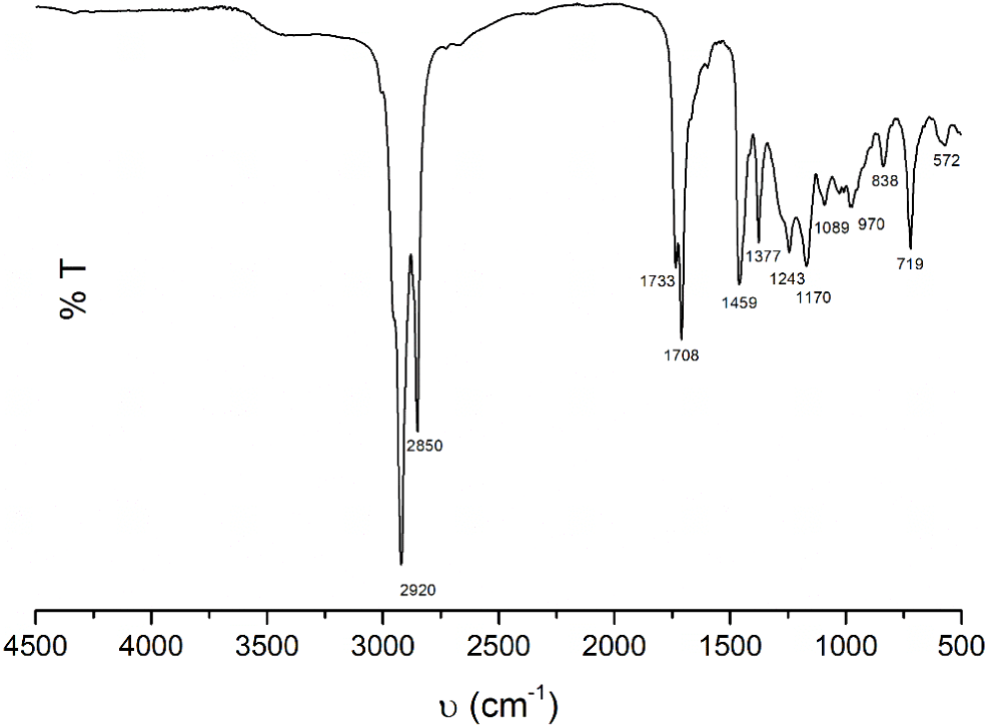
FTIR spectrum of *M. frigidus* hexanic extract (MFH) in transmittance mode.

### 3.2 *In Vitro* Antifungal Activity of *Mitracarpus frigidus* Hexanic Extract (MFH)

Table 2 shows the MIC and MFC values of MFH against *Candida* spp. strains. MFH showed MIC values between 250 and 1000 μg/mL, highlighting all the *C. albicans* with MIC value of 250 μg/mL, followed by MIC values of 500 and 1000 μg/mL against *C. glabrata* and *C. krusei*. MFH showed MFC value of 500 μg/mL for *C. albicans* vv3 and *C. albicans* mo2, 1000 μg/mL for *C. albicans* ATCC 10231 and *C. albicans* vv1. For the other strains, MFC could not be determined at the concentrations tested. The MIC values for fluconazole and nystatin were 1000–31.25 μg/mL and 2.5 μg/mL, respectively. These results showed a resistance to *Candida* strains tested, except *C. albicans* ATCC 10231 and *C. krusei* for fluconazole and a sensibility for nystatin. Considering that *Candida* strains with a MIC ≥ 64 μg/mL are classified as resistant to fluconazole.^61^

**Table 2:** Minimum Inhibitory Concentration (MIC) and Minimum Fungicidal Concentration (MFC) of *Mitracarpus frigidus* Hexane Extract (MFH) Against *Candida* Species^a^

	MFH			
Microorganisms	MIC values (μg/mL)	MFC values (μg/mL)	Nystatin MIC values (μg/mL)	Fluconazole MIC values (μg/mL)
*C. albicans*. ATCC 24433	250	>1000	2.5	1000
*C. albicans* ATCC 10231	250	1000	2.5	31.25
*C. glabrata* ATCC 2001	500	>1000	2.5	125
*C. krusei* ATCC 6258	1000	>1000	2.5	31.25
*C. albicans* vv1	250	1000	2.5	1000
*C. albicans* vv2	250	>1000	2.5	1000
*C. albicans* vv3	250	500	2.5	1000
*C. albicans* mo2	250	500	2.5	250
*C. albicans* mo4	250	>1000	2.5	250

a Nystatin and fluconazole were used as positive controls.

Azoles are the most frequently prescribed antifungal agents for the treatment of *C. albicans* infections.^62^ However, the increasing occurrence of azole-resistant strains has limited their effectiveness.^63^ Consequently, alternative therapeutic strategies are needed. Plant extracts exhibiting MIC values below 1000 μg/mL are generally regarded as promising antimicrobial candidates.^57,59^ In this context, MFH demonstrated notable antifungal activity in our assay and could represent a potential therapeutic approach for managing candidiasis, particularly in cases involving fluconazole-resistant *C. albicans* *strains*. Furthermore, a fungistatic effect was observed at all MIC values, indicating the ability of the extract to inhibit fungal growth in various *Candida* species. Our results showed MIC values similar to those of other nonpolar phytochemicals, such as oils extracted from both ripe and unripe fruits of Protium ovatum, which exhibited fungistatic effects with MIC values ranging from 250 to 62.5 μg/mL.^64^

*Candida* species are among the five leading causes of hospital-acquired infections and the four leading causes of systemic infections.^65^ Infections caused by *Candida* spp. infections are common and can be both noninvasive, such as oral and vulvovaginal mucosal infections, and invasive, affecting the bloodstream and deep tissues.^66^ In intensive care units, invasive infections are on the rise and *Candida* species are responsible for around 80% of these infections.^59^ Despite the increasing incidence of non-*albicans* *Candida* (NAC) infections, *C. albicans* remains the most commonly observed species compared to those associated with NAC infections, as reported by several epidemiologic studies in the world.^62,67^ One approach in the search for new drugs is the investigation study of natural products, which are considered promising sources of bioactive compounds with potential for disease treatment.^68,69^ Therefore, the evaluation of the antifungal activity of MFH has remarkable clinical relevance and is the focus of this work.

#### 3.2.1 Fungal Growth Curves

Fungal growth curves of *C. albicans* ATCC 24433, *C. albicans* ATCC 10231, and *C. albicans* vv1 in the presence of MFH and nystatin (MIC values) were analyzed for 48 h and are shown in Figure 3. MFH altered cell growth in *Candida* strains tested. Figure 3Ai shows that MFH and nystatin treatments induced an increase in the lag phase (adaptation period) of *C. albicans* ATCC 24433 when compared to the growth control. Cells treated with MFH and nystatin exhibited a lag phase up to 24 and 12 h, respectively, while untreated yeast (growth control) exhibited a lag phase up to 4 h. In addition, there was a significant reduction in the log phase (growth time) of yeast in the MFH/nystatin treatment compared to the growth control (*p* < 0.05). There was a statistical difference between the treatments and the growth control as shown by the area under the curve of the 48 h experiment (Figure 2A) (*p* < 0.05).

**Figure 3:**
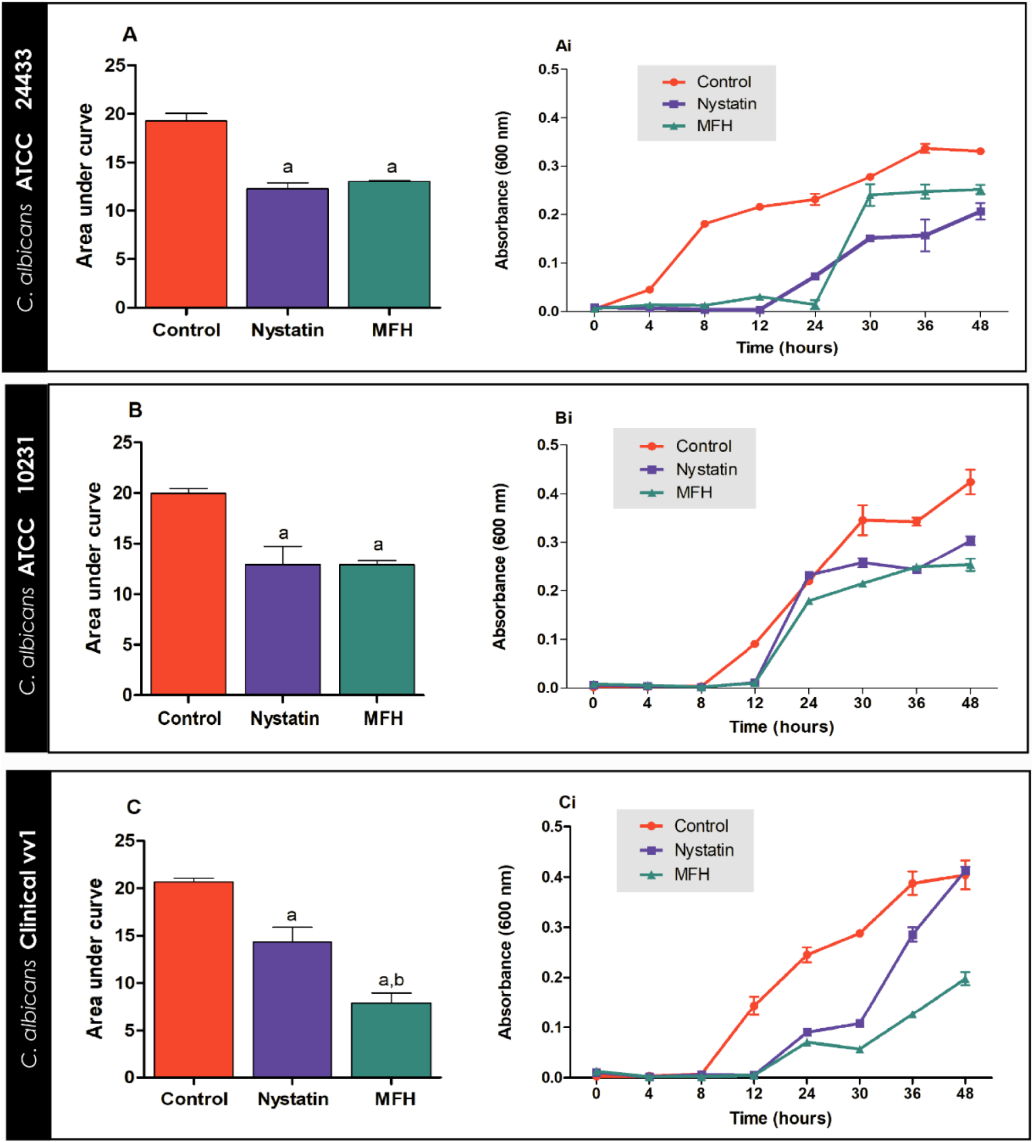
Time-kill kinetics of *M. frigidus* hexane extract (MFH) against *C. albicans* ATCC 24433 (A), *C. albicans* ATCC 10231 (B), and *C. albicans* vv1 (C). Nystatin was used as a positive control and the MFH vehicle was used as fungal growth control. (A, B, and C) Represent the growth curves of control and treatment groups and (Ai, Bi, and Ci), the area under the curve. Letters indicate statistical differences from groups treated with control (a) and nystatin (b) (ANOVA followed by Bonferroni, *p* < 0.05). All experiments were conducted in triplicate.

Figure 3Bi shows an increased lag phase of *C. albicans* ATCC 10231 after MFH and nystatin treatment compared to the growth control. The untreated yeasts showed a lag phase up to 8 h, while the yeasts treated with MFH and nystatin showed a lag phase of up to 12 h. Furthermore, the log phase of the MFH/nystatin-treated yeast was significantly reduced compared to the untreated yeast (*p* < 0.05). From the area under the graph of the experiment at 48 h (Figure 3B), the treatments were statistically different from the growth control (*p* < 0.05).

An increase in the lag phase of *C. albicans* vv1 was also observed after treatment with MFH and nystatin compared to the control, as shown in Figure 3Ci. Untreated yeasts exhibited a lag phase up to 8 h, while those treated with MFH and nystatin had a lag phase up to 12 h. Yeasts treated with MFH and nystatin showed a significant reduction in the log phase compared to untreated yeasts. The area under the curve plot of the 48-h experiment (Figure 3C) showed a statistically significant difference between MFH and nystatin treatments (*p* < 0.05). Moreover, MFH and treatments showed significant differences when compared to controls (*p* < 0.05).

The fungal growth curve allowed evaluation of the behavior of three *C. albicans* strains when exposed to MFH and nystatin (MIC values). MFH and nystatin acted mainly in the lag and log phases of the fungal growth curve. These results suggest that MFH and nystatin interfere with fungal adaptation to the environment (lag phase), when there is intense metabolic activity but no population growth. MFH and nystatin also interfered with the log phase, which is characterized by intense population growth and metabolic activity, as shown by the area under the curve graph for each strain. Inhibiting fungal growth is crucial for controlling infections, as restricting the proliferation of rapidly growing fungi can help prevent disease development in susceptible individuals.^70,71^

#### 3.2.2 Effect of *Mitracarpus frigidus* Hexane Extract (MFH) on Fungal Cell Density

Fungal cells were stained with CW (Calcofluor White) and quantified by fluorescence microscopy to evaluate the effect of MFH on the cell density of *C. albicans* strains. Results were expressed as cell counts × 10^6^ cells/mL (Figure 4). It was possible to observe that MFH reduced the cell density of *C. albicans* ATCC 24433, *C. albicans* ATCC 10231, and *C. albicans* vv1 by 60, 63.5, and 57%, respectively, compared to controls (*p* < 0.05). However, nystatin reduced the cell viability of *C. albicans* ATCC24433, *C. albicans* ATCC10231, and *C. albicans* clinical vv1 by 60, 52, and 57%, respectively, compared to controls (*p* < 0.05).

**Figure 4:**
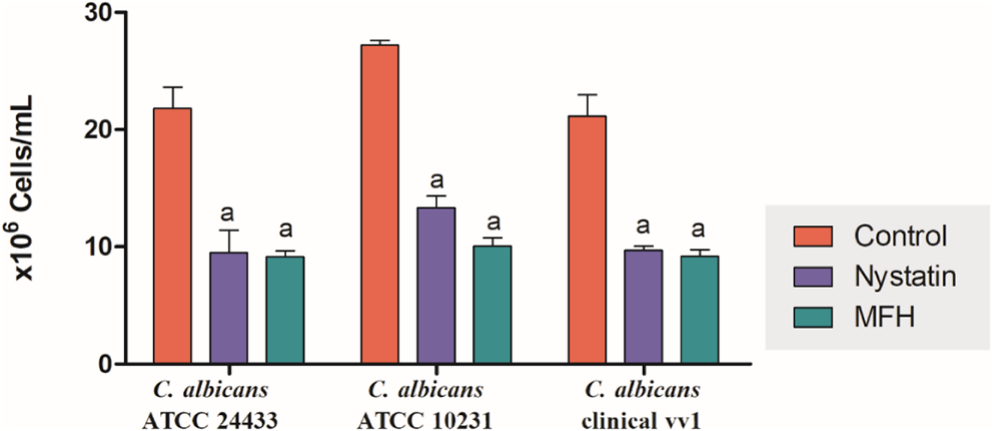
Activity of *M. frigidus* hexane extract (MFH) on fungal cell density of *C. albicans* ATCC 24433, *C. albicans* ATCC 10231, and *C. albicans* vv1. Fungal cultures were stained with DAPI and counted under fluorescence microscopy for evaluation of cell density. *M. frigidus* hexane extract (MFH) treatment induced a decrease in cell density. Nystatin was used as a positive control and the MFH vehicle was used as fungal control. Letters indicate statistical differences from group treated with control (a) (ANOVA followed by Bonferroni, *p* < 0.05). All experiments were conducted in triplicate, and data are presented as the mean ± SD of fungal counts.

The CW dye used is a fluorescent marker that, when bound to the chitin of the fungal cell wall, increases its fluorescence and emits a blue color.^72,73^ MFH and nystatin similarly reduces the cell density of *Candida* spp. strains compared to the control (*p* < 0.05), indicating a reduction in fungal burden and confirming the results of MIC and growth curve assays. Campos et al.^15^ showed that antifungal activity of the methanolic extract of *M. frigidus* was also observed against *C. albicans* ATCC 10231 through the same assays, which reinforces the antifungal potential of the plant species.

#### 3.2.3 Effect of *Mitracarpus Frigidus* Hexane Extract (MFH) on the Fungal Cell Envelope

In the cell envelope analyses, the experiments performed were designed to evaluate the effect of MFH on the fungal cell membrane. Figure 5 indicate that the percentage of violet crystal taken up by *C. albicans* ATCC 24433, *C. albicans* ATCC 10231, and *C. albicans* vv1 strains was increased by 5.6, 12.4, and 8.3 times, respectively (*p* < 0.05) in MFH-treated cells compared to control. However, nystatin-treated fungal cells showed a 4.4-, 9.4-, and 6.0-fold increase in the rate of violet crystal uptake by *C. albicans* ATCC 24433, *C. albicans* ATCC 10231, and *C. albicans* vv1 strains, respectively, compared to controls (*p* < 0.05). Furthermore, there was a statistical difference in crystal violet uptake by MFH-treated cells compared to nystatin-treated cells (*p* < 0.05).

**Figure 5:**
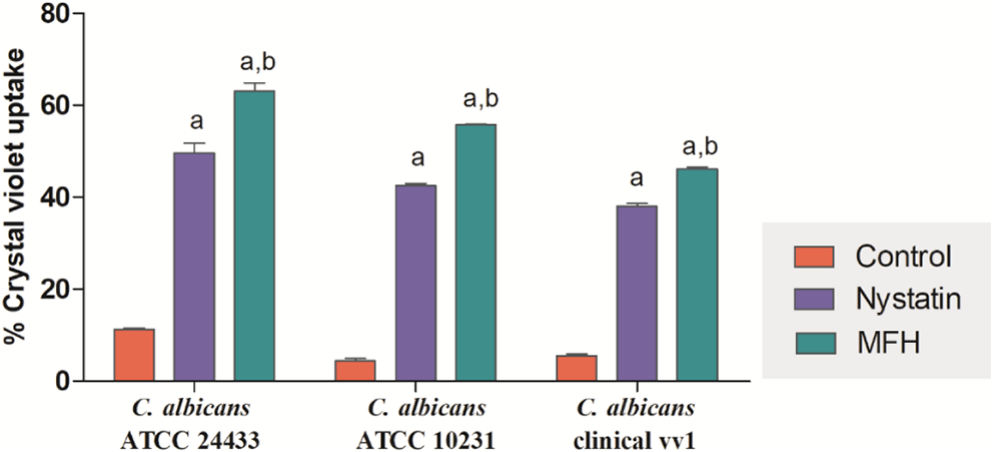
Measurement of cell permeability by percentage of crystal violet uptake from *C. albicans* ATCC 24433, *C. albicans* ATCC 10231, and *C. albicans* vv1. Cultures treated with *M. frigidus* hexane extract (MFH) or nystatin (positive control) were assessed for cell membrane permeability using the crystal violet assay. The control group received the MFH vehicle. Letters indicate statistically significant differences from the control group (a) and nystatin-treated group (b) (ANOVA followed by Bonferroni post hoc test, *p* < 0.05). Experiments were performed in triplicate, and data are presented as mean ± SD.

#### 3.2.4 Effect of *Mitracarpus frigidus* Hexane Extract (MFH) in Fungal Cell Membrane Permeability

The violet crystal assay can detect changes in cell membrane permeability. The uptake of crystal violet by the cell is slightly increased when the cell envelope is damaged. This uptake is lower when the envelope is intact.^15^ MFH had a significant effect on the membrane permeability of the three *Candida* strains when compared to nystatin (*p* < 0.05).

The membrane leakage and protein content assays allow the effect of MFH and nystatin treatments on the cell permeability of fungal cells to be evaluated (Figure 6). Figure 6Ai shows that MFH treatment altered the cell permeability of *C. albicans* ATCC 24433, resulting in greater nucleotide extravasation compared to nystatin treatment and control, mainly after the first hour of treatment. Figure 6A shows a 12-fold increase in the area under the curve of MFH-treated yeast relative to the control (*p* < 0.05). In contrast, the area under the curve of the yeast treated with nystatin shows a 7-fold increase concerning the control (*p* < 0.05). MFH treatment increased protein levels by 15.2-fold over control (*p* < 0.05, Figure 6Aii). Nystatin treatment had a 13.8-fold increase in protein level over control (*p* < 0.05, Figure 6Aii). There was also a statistical difference observed between the MFH treatment and the nystatin treatment in both assays (*p* < 0.05).

**Figure 6:**
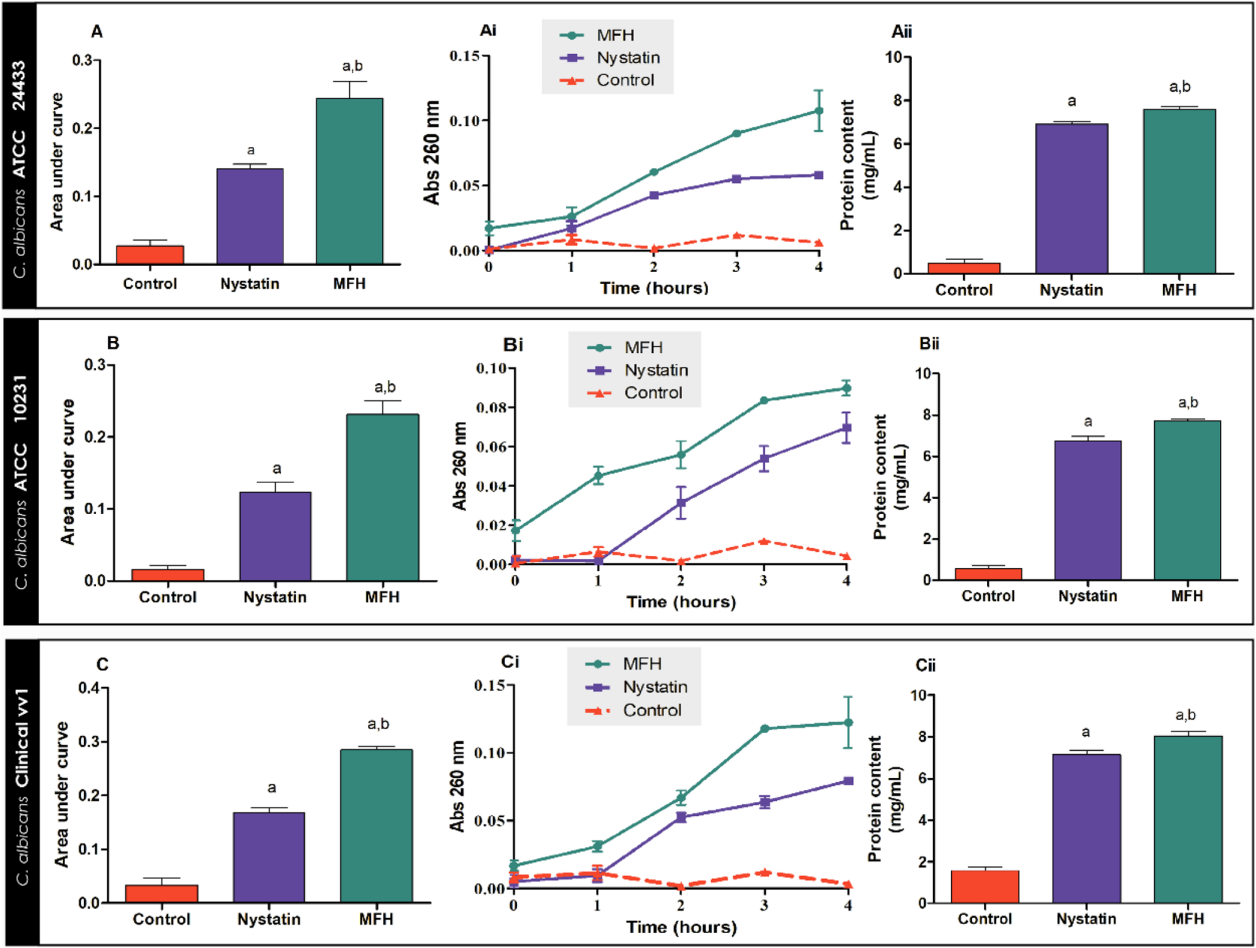
Nucleotide and protein release from *C. albicans* ATCC 24433 (A), *C. albicans* ATCC 10231 (B), and *C. albicans* vv1 (C) after treatment with *M. frigidus* hexane extract (MFH), MFH vehicle (control) and nystatin, positive control. (A, B, and C) Represents the nucleotide extravasation curves measured by optical density at 260 nm for 4 at 1-h intervals; (Ai, Bi, and Ci) Area under curve; (Aii, Bii, and Cii) Protein content after 4 h of MFH and nystatin treatments. Letters indicate statistical differences from groups treated with control (a) and nystatin (b) (ANOVA followed by Bonferroni, *p* < 0.05). All experiments were conducted in triplicate, and the results are expressed as mean ± standard deviation (SD).

Figure 6Bi results showed that MFH also altered *C. albicans* ATCC 10231 cell permeability, with greater nucleotide extravasation compared to nystatin treatment and control. The area under the curve (Figure 6B) referring to the yeasts treated with MFH exhibited a 15.4-fold increase compared to the control, while that referring to the nystatin treatment had an 8.0-fold increase over the control (*p* < 0.05). An increase in protein content was also observed in the MFH and nystatin-treated yeasts compared to the control. MFH treatment caused a 12.8-fold increase in protein content compared to the control, while nystatin caused an 11.3-fold increase relative to the control (*p* < 0.05, Figure 6Bii). MFH treatment and nystatin treatment were statistically different in both tests (*p* < 0.05).

MFH also affected the cell permeability of *C. albicans* vv1 (Figure 6Ci), allowing greater nucleotide extravasation compared to nystatin treatment and control. Figure 6C shows that the area under the curve of MFH-treated yeasts increased 8.6-fold relative to control, whereas nystatin-treated yeasts increased 5.0-fold relative to control (*p* < 0.05). In terms of protein content, a 5.1-fold increase was observed after MFH treatment compared to that observed in the control (*p* < 0.05, Figure 6Cii). In contrast, nystatin treatment increased protein levels 4.5-fold over the control (*p* < 0.05, Figure 6Cii). A statistical difference between MFH and nystatin treatment was also observed in both studies (*p* < 0.05).

The effect of MFH on fungal cell permeability was evaluated based on the release of intracellular contents into the medium.^74^ The results showed that MFH had a better ability to extract intracellular contents from the three *Candida* strains compared to nystatin. Thus, MFH induced greater damage to the cell membrane, resulting in increased extravasation of cellular contents compared to the respective controls (*p* < 0.05).

#### 3.2.5 Effects of MFH Extract on the Sorbitol Protection and Ergosterol Binding

Sorbitol protection and exogenous ergosterol binding assays allowed us to analyze the effect of MFH and nystatin on the cell wall via sorbitol protection and in the cell membrane via exogenous ergosterol binding. These results showed an increase in MIC value in the presence of ergosterol (1000 and 2.5 μg/mL, respectively) and no change in MIC value in the presence of sorbitol (250 and 10 μg/mL, respectively) against the *C. albicans* strains tested (Table 3).

**Table 3:** Minimum Inhibitory Concentration (MIC) of *Mitracarpus frigidus* Hexane Extract (MFH) Against *Candida albicans* Species in the Presence and Absence of Sorbitol and Exogen Ergosterol for the Microtiter Dilution Broth

Microorganisms	MIC values of MFH (μg/mL)	MIC values of MFH with sorbitol (μg/mL)	MIC values of MFH with ergosterol (μg/mL)	MIC values of nystatin (μg/mL)	MIC values of nystatin with sorbitol (μg/mL)	MIC values of nystatin with ergosterol (μg/mL)
*C. albicans* ATCC 24433	250	250	1000	2.5	2.5	10
*C. albicans* ATCC 10231	250	250	1000	2.5	2.5	10
*C. albicans* vv1	250	250	1000	2.5	2.5	10

Sorbitol is an osmotic protector that promotes the growth of fungal cells, even in the presence of compounds that cause damage to the essential components of the cell wall and consequent cell lysis. Therefore, sorbitol in the medium will allow growth if a sample reacts with the fungal cell wall, increasing the MIC value.^75^ Thus, if a sample acts on the fungal cell wall, the presence of sorbitol in the medium will allow growth, leading to an increase in the MIC value.^32^ In the case of MFH, the MIC values remained the same for all the strains tested, which means that MFH does not act directly on the cell wall, confirming its fungistatic effect (Table 3).

Ergosterol is an essential sterol in fungal membranes, playing a key role in maintaining membrane structure, rigidity, permeability, and the proper function of membrane proteins. Its biosynthesis is closely linked to antifungal resistance and the virulence of pathogenic fungi.^76^ Due to its crucial role in enabling fungi to adapt to abiotic stresses, ergosterol has become a key target for various antifungal drugs, including nystatin and azoles.^77^ When these drugs bind to the membrane, they form large extramembranous aggregates that extract ergosterol from the lipid bilayers, resulting in cell lysis.^76^ Moreover, azoles inhibit sterol demethylase, a critical enzyme in the ergosterol biosynthetic pathway.^78^ In the assay, exogenous ergosterol is introduced into the culture medium. If the sample exhibits an affinity for this exogenous ergosterol, it will tend to bind to it, causing a shift in drug activity and resulting in an elevated MIC value.^15,79^

MFH, as well as nystatin (positive control), showed increased MIC values for all *Candida* strains, demonstrating the action of these two compounds on this fungal membrane component. To analyze the interaction of the samples with ergosterol, fungal cells of each strain were treated with MFH and nystatin (MIC values), and the ergosterol content after extraction was analyzed. Figure 7 shows that the treatments induced an increased percentage of extracted ergosterol in *C. albicans* strains compared to the control (*p* < 0.05). Moreover, nystatin, a polyene antifungal, showed a higher extraction of ergosterol when compared to MFH (*p* < 0.05).

**Figure 7:**
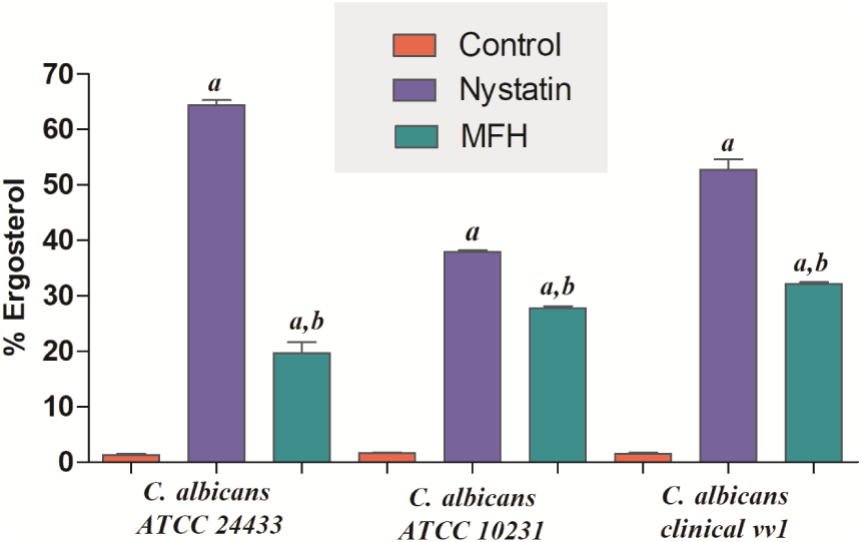
Percentage of ergosterol extracted after treatment with *M. frigidus* hexane extract (MFH) and nystatin, positive control. Fungal cell cultures were treated with MFH and nystatin and, after extraction, the ergosterol content was analyzed. Different letters denote statistically significant differences compared to the control group (a) and the nystatin-treated group (b), as determined by ANOVA followed by Bonferroni’s post hoc test (*p* < 0.05). Experiments were conducted in triplicate, and data are presented as mean ± SD.

The determination of ergosterol content is consistent with previous findings, as this assay allows us to assess whether MFH can interact with membrane ergosterol, extract it from the cells, or disrupt ergosterol biosynthesis, thereby reducing its levels inside the cells.^34,80^ It was observed that MFH increased the extravasation of ergosterol in the *Candida* strains, indicating that an interaction with membrane ergosterol occurs without interfering with biosynthesis.

#### 3.2.6 Action of *Mitracarpus frigidus* Hexane Extract (MFH) on the Mitochondrial Membrane Potential

Mitochondria-focused studies were conducted to further substantiate and validate the antifungal activity of MFH. The treatment with MFH resulted in a distinct change in the dispersion profile of fungal cells compared to the control (Figure 8). Both MFH and nystatin induced mitochondrial membrane depolarization, resulting in mitochondrial inactivation. The reduction in fluorescence intensity in the MFH and nystatin treatments was approximately 79 and 81%, respectively, compared to the control group (*p* < 0.05).

**Figure 8:**
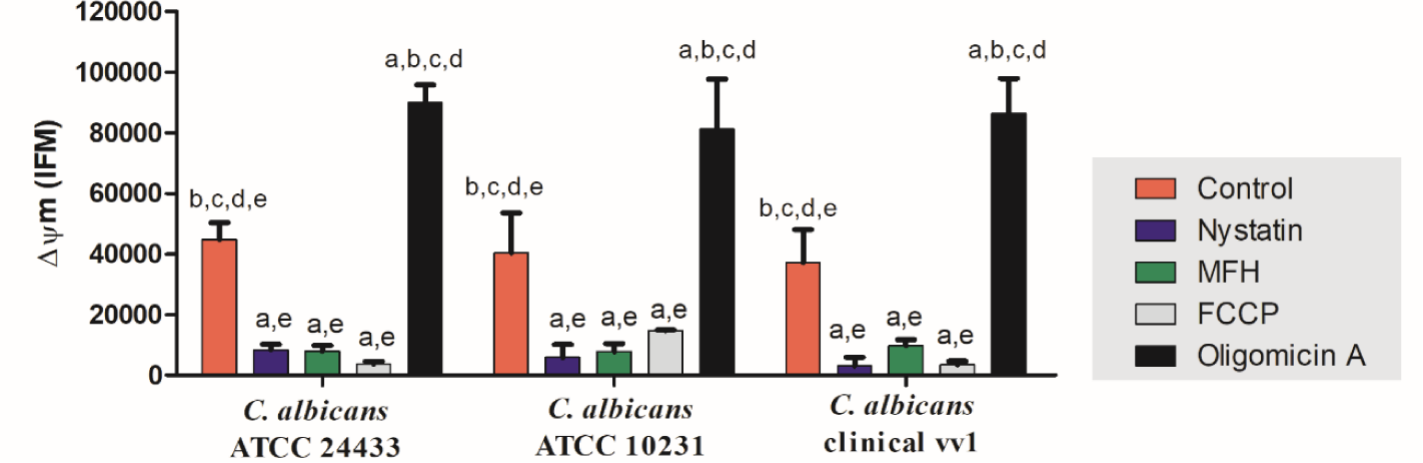
Action of *M. frigidus* hexane extract (MFH) and nystatin, positive control, on the mitochondrial membrane potential of *C. albicans* ATCC 24433, *C. albicans* ATCC 10231, and *C. albicans* vv1. Graph showing the fluorescence intensity of cells treated with MFH, nystatin, FCCP, and oligomycin A after TMRE staining. Different letters indicate statistically significant differences relative to the control (a), nystatin (b), MFH (c), FCCP (d), and oligomycin A (e), as determined by ANOVA followed by Bonferroni’s post hoc test (*p* < 0.05). Experiments were performed in triplicate, and data are presented as mean ± SD.

Mitochondria play a crucial role in generating the mitochondrial membrane potential by pumping protons across the inner membrane, resulting in a high potential energy gradient.^35^ Experimental findings with fungal cells treated with MFH and nystatin indicated reduced membrane potential and diminished binding of TMRE, indicating mitochondrial depolarization induced by the treatments. TMRE exhibits a significant impact on antifungal activity, affecting both virulence and the cell envelope of *C. albicans*.

#### 3.2.7 Effect of *Mitracarpus frigidus* Hexane Extract (MFH) on Fungal Cell Viability

The LIVE/DEAD BacLight kit was used to analyze the cell viability of *C. albicans* strains treated with MFH and nystatin (Figure 9). Figure 9A shows illustrative images of the assay, emphasizing fungal cells (treated and untreated) stained with Syto 9, one of the dyes in the kit, which stains all cells that are intact or with membrane damage green. In contrast, propidium iodide (PI), the other dye in the kit, penetrates only into cells with membrane damage, causing a reduction in the fluorescence of Syto 9 and staining the cells red. Figure 9B, in turn, presents the percentage of viable and nonviable cells after MFH and nystatin treatments.

**Figure 9:**
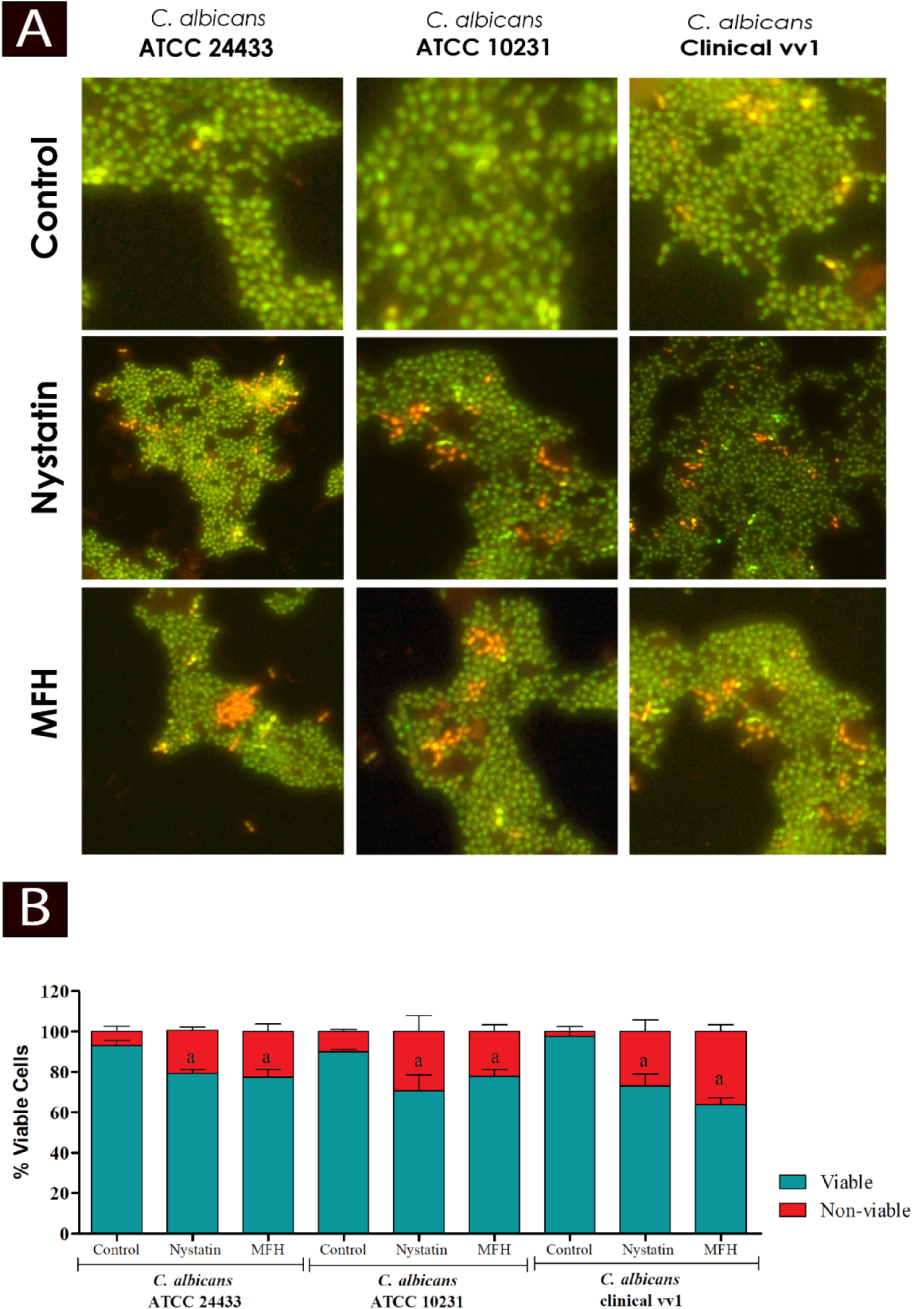
Effect of *M. frigidus* hexane extract (MFH) on fungal cell viability of *C. albicans* ATCC 24433, *C. albicans* ATCC 10231, and *C. albicans* vv1. Fungal cultures were stained with LIVE/DEAD BacLight and analyzed under fluorescence microscopy to assess cell viability. MFH treatment increased cell death (B) compared to the control group treated with MFH vehicle. Panel (A) shows representative images of treated and untreated fungi after BacLight staining, with viable cells appearing green and nonviable cells red. An increase in dead cells was observed following MFH treatment. Letters indicate statistically significant differences relative to the control (a) and nystatin, positive control (b) (ANOVA followed by Bonferroni’s post hoc test, *p* < 0.05). Experiments were performed in triplicate, and data are presented as mean ± SD. Scale bar: 10 μm.

The results showed that *C. albicans* ATCC 24433 treated with MFH and nystatin had a percentage of nonviable cells of 22 and 20%, respectively, while the control had a percentage of 7%. *C. albicans* ATCC 10231 treated with MFH showed 22% of nonviable cells. The percentage of nonviable cells of the yeasts treated with nystatin was 30%, versus 10% for the control. *C. albicans* vv1 treated with MFH and nystatin showed 37 and 27% nonviable cells, respectively, compared to 2% nonviable cells in the control. MFH and nystatin treatments were statistically different when compared from controls (*p* < 0.05).

### 3.3 In Vitro Cytotoxicity Assessment

Furthermore, the cytotoxic potential of MFH on mammalian cells was assessed using peritoneal macrophages from BALB/c mice and the L929 fibroblast cell line (Figure 10A-B, respectively). According to ISO 2009 guidelines, a cell viability of 70% or higher is considered noncytotoxic. Under these criteria, all tested concentrations of MFH were found to be noncytotoxic (*p* < 0.05). For new drug therapies to be developed and incorporated, they must be effective with low or no cytotoxicity to mammalian cells, making them targets for further investigation in nonclinical models of in vivo toxicity.^81^

**Figure 10:**
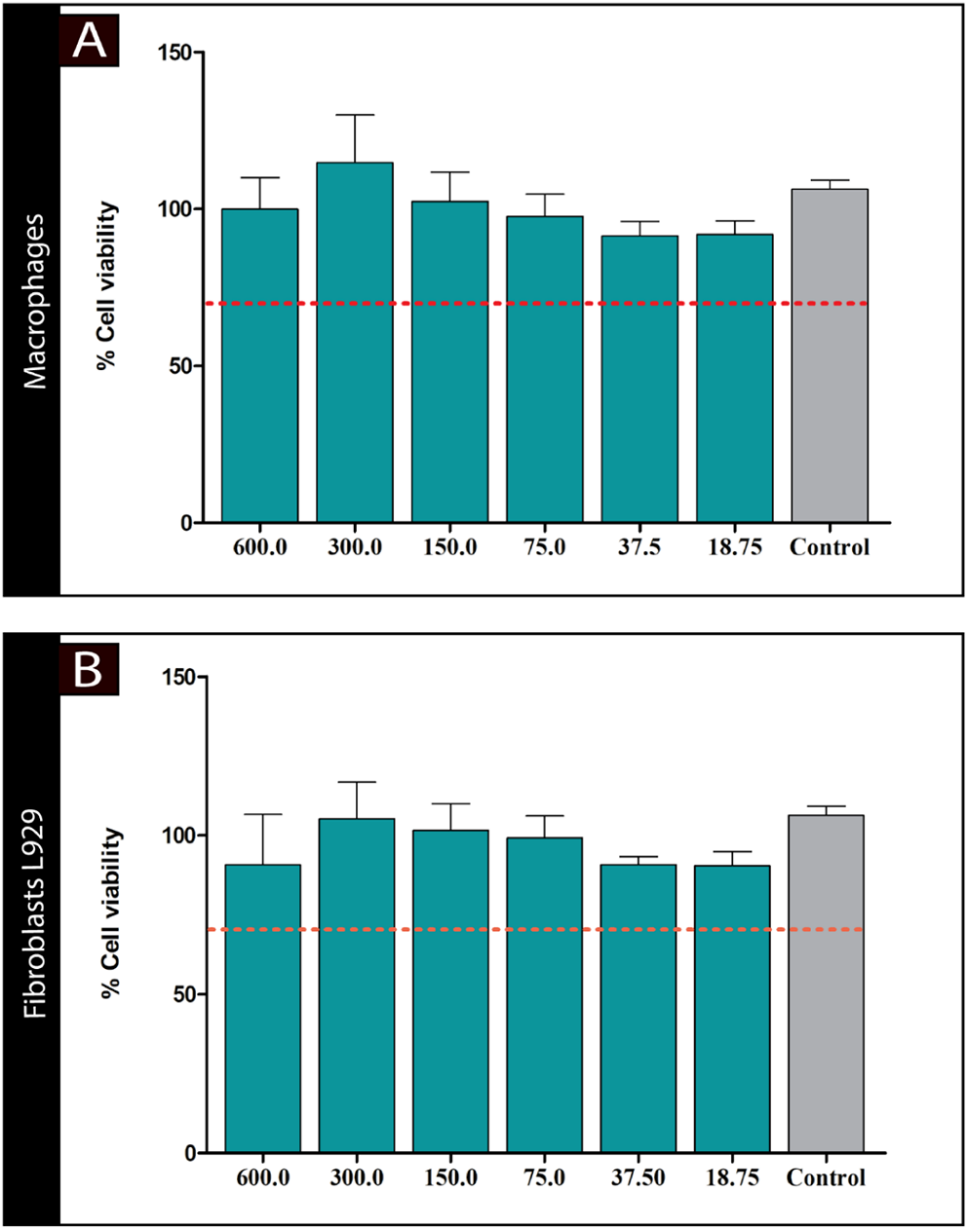
Cytotoxicity of *M. frigidus* hexane extract (MFH) against peritoneal macrophages from BALB/c mice (A) and fibroblasts L929 (B) cells after 48 h of exposure. Mammalian cells were incubated with different concentrations of the complexes (18.75 to 600 μg/mL). The cell viability was determined by the MTT colorimetric method. The result was expressed as the percentage of survival in relation to untreated cells. The red dashed line indicates the cell viability percentage of 70%. All experiments were performed in triplicate.

### 3.4 *In Vivo* Antifungal Activity in the Vulvovaginal Candidiasis Model

Based on our in vitro findings suggesting antifungal potential, in vivo experiments were conducted to evaluate the therapeutic effect of MFH in an experimental model of vulvovaginal candidiasis (VVC). To achieve rapid fungal colonization with persistent mucosal infection, the model requires systemic immunosuppression. In rodents, cyclophosphamide inhibits antibody production and targets rapidly dividing cells, suppressing both humoral and cellular immune responses.^82^ Since local immunity is critical for defense against *Candida* in the vaginal mucosa, estrogen treatment was also administered to induce a pseudoestrous phase, impair local immune defenses, and increase glycogen content, thereby facilitating mucosal infection.^83^ Under these conditions, intravaginal instillation of *C. albicans* successfully established infection in the hormonal-induced, immunosuppressed rats.

Following infection establishment, the therapeutic effects of three MFH concentrations and clotrimazole (CTZ) were evaluated. On day 6 of treatment, fungal loads remained significantly elevated in the negative and vehicle control groups (Table 4). In contrast, MFH at 0.5 and 1.0 mg/mL produced substantial reductions in fungal burden, achieving approximately 80% and 95% inhibition, respectively. As expected, CTZ treatment completely eradicated *C. albicans* by the final day of the experiment.

**Table 4:** Number of Infected Animals and Corresponding Vaginal Fungal Load in Wistar Rats Treated Intravaginally with Different Concentrations of *Mitracarpus frigidus* Hexane Extract (MFH) and Respective Controls

	Day 1		Day 3		Day 6		
Groups	Infected animals/N (%)	Log CFU/mL ± SD	Infected animals/N (%)	Log CFU/mL^1^ ± SD	Infected animals/N (%)	Log CFU/mL ± SD	% of reduction^a^
Infected control	6/6 (100)	4.69 ± 0.20	6/6 (100)	4.65 ± 0.84	6/6 (100)	4.24 ± 0.42	9.59
CTZ	6/6 (100)	3.89 ± 0.590	3/6 (50)	2.71 ± 0.660	0/6 (0)	0 ± 0^b^	100
MFH 0.1 mg/mL	6/6 (100)	3.94 ± 0.11	6/6 (50)	3.94 ± 0.10	3/6 (50)	2.32 ± 1.05^b^	41.11
MFH 0.5 mg/mL	6/6 (100)	4.04 ± 0.05	6/6 (30)	3.99 ± 0.08	1/6 (10)	0.77 ± 0.98^b^	80.94
MFH 1.0 mg/mL	6/6 (100)	4.01 ± 0.09	6/6 (30)	3.96 ± 0.09	1/6 (10)	0.19 ± 0.45^b^	95.26
Vehicle	6/6 (100)	4.06 ± 0.05	6/6 (100)	4.04 ± 0.03	6/6 (100)	4.64 ± 0.37	-

a CTZ- clotrimazole cream. Percentage reduction of fungal load relative to day 1 of treatment.

b Indicates a statistically significant difference compared to day 1 (ANOVA followed by Bonferroni, *p* < 0.05).

The noninfected control group displayed minimal infiltration of inflammatory cells and no detectable fungal organisms in the vaginal mucosa (Figure 11A). In contrast, the infected control group exhibited moderate mucosal inflammation accompanied by fungal organisms within the stratum corneum (Figure 11B). Interestingly, animals treated with clotrimazole presented variable inflammation ranging from mild to severe, along with numerous to abundant fungal organisms, sometimes exceeding the levels observed in the infected control group (Figure 11C). In the MFH-treated groups, inflammation ranged from mild to severe, but no fungal organisms were detected (Figure 11E–G). Other histological alterations were primarily localized to the vaginal mucosa, including mild to moderate hyperkeratosis and hyperplasia (acanthosis). The acanthotic epithelium displayed cytoplasmic vacuolization, pyknosis, and loss of keratinocytes (Figure 11). These degenerative and cell death changes were most evident in infected animals treated with clotrimazole (Figure 11C) or vehicle control (Figure 11D), whereas keratinocyte degeneration was less pronounced in MFH-treated animals. Multifocal to coalescing inflammatory infiltrates expanded the lamina propria/submucosa and consisted predominantly of neutrophils and macrophages, with fewer lymphocytes.

**Figure 11:**
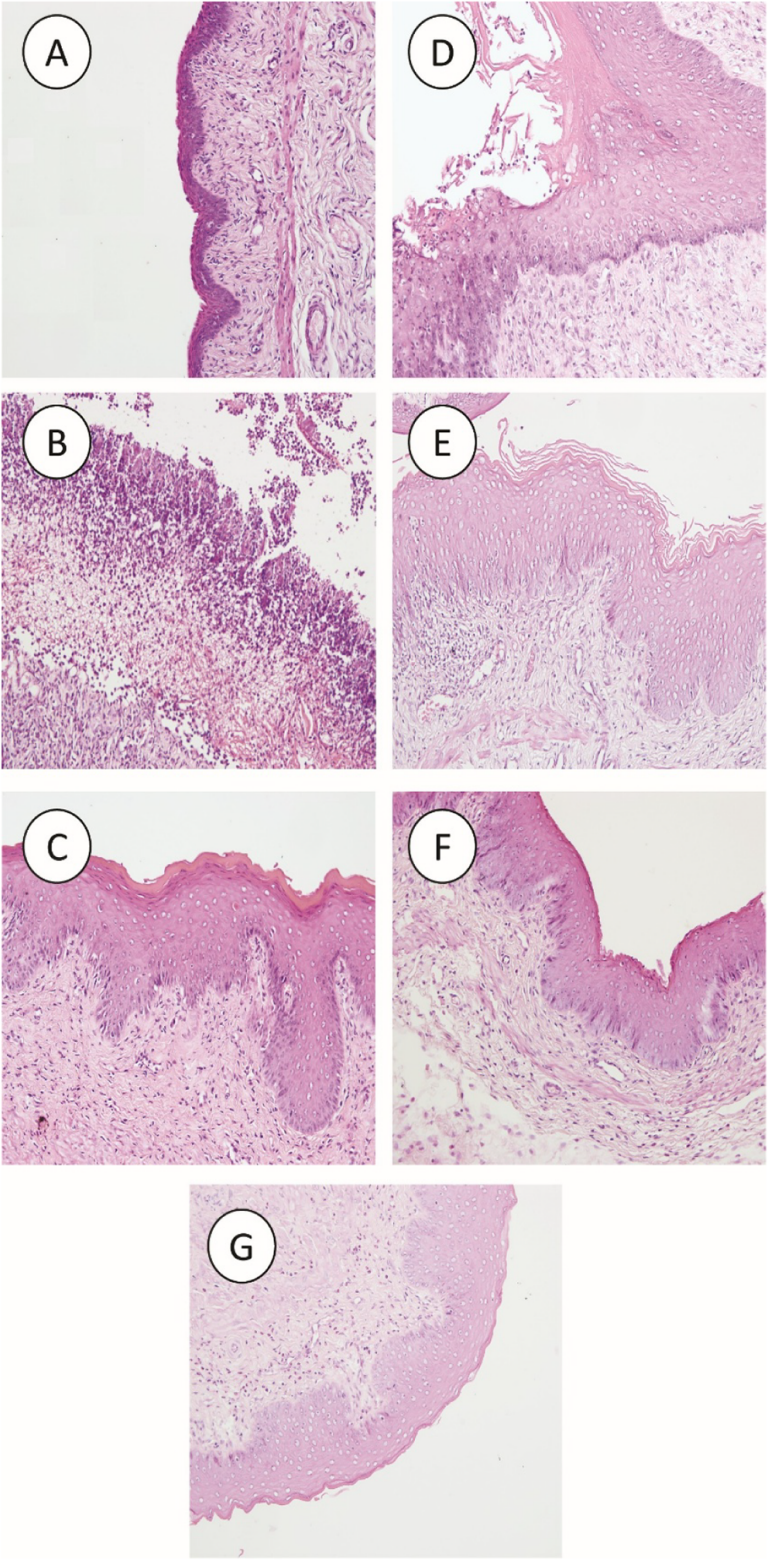
Representative photomicrographs of longitudinal vaginal sections stained with Hematoxylin and Eosin. (A) Noninfected control animals; (B) Infected, nontreated animals (negative control); (C) Infected animals treated with clotrimazole cream (positive control, 10 mg/g); (D) Infected animals treated with MFH vehicle; (E) Infected animals treated with MFH 0.1 mg/mL; (F) Infected animals treated with MFH 0.5 mg/mL; (G) Infected animals treated with MFH 1.0 mg/mL. Magnification, 200×; scale bar, 100 μm.

The antifungal activity of MFH observed in both in vitro and in vivo assays may be related to the presence of bioactive compounds, including pentalongin, squalene, neophytadiene, stigmasterol, γ-sitosterol, and 2-azaanthraquinone. It is likely that a synergistic interaction among these constituents contributes to the overall antifungal efficacy of MFH.

## 4 Conclusion

This study shows that *M. frigidus* has promising potential as an antifungal ingredient for the pharmaceutical industry. For the first time, it demonstrated that MFH and its bioactive molecules exhibit promising antifungal activity without showing cytotoxicity in peritoneal macrophages from BALB/c mice or L929 fibroblasts. The antifungal activity of MFH was similar for all *Candida* species investigated, regardless of their resistance to commercially available antifungals. This indicates a fungistatic effect and suggests that MFH acts mainly on the fungal cell envelope by binding to ergosterol. This disrupts the mitochondrial membrane potential, compromises membrane integrity, and causes leakage of intracellular contents. In vivo, MFH exhibited no cytotoxic effects, effectively reduced the fungal load in vaginal lavage, and prevented fungal penetration into the stratum corneum, thereby mitigating the inflammatory response associated with tissue invasion. Together, these results confirm the preclinical potential of MFH in treating fungal infections caused by *Candida* species, particularly vulvovaginal candidiasis.

## Supplementary Material



## Data Availability

All the data from this work are available in the paper. The authorization for the use of the plant species is registered in SISGEN/Brazil-A032F41.

## Notes

Gas chromatography-mass spectrometry (GC-MS) profile of *Mitracarpus frigidus* hexane extract (MFH) (PDF)
