# Supplementary material for: Mitracarpus frigidus in the Treatment of Vulvovaginal Candidiasis: A Comprehensive Evaluation of Its Therapeutic Properties
Source: ACS Omega. 2025 Oct 17;10(42):49828–44. doi: 10.1021/acsomega.5c05713 (PMC12573053; doi:10.1021/acsomega.5c05713)

Supplementary Material

S1- Gas chromatography-mass spectrometry (GC-MS) profile of *Mitracarpus frigidus* hexane extract (MFH).

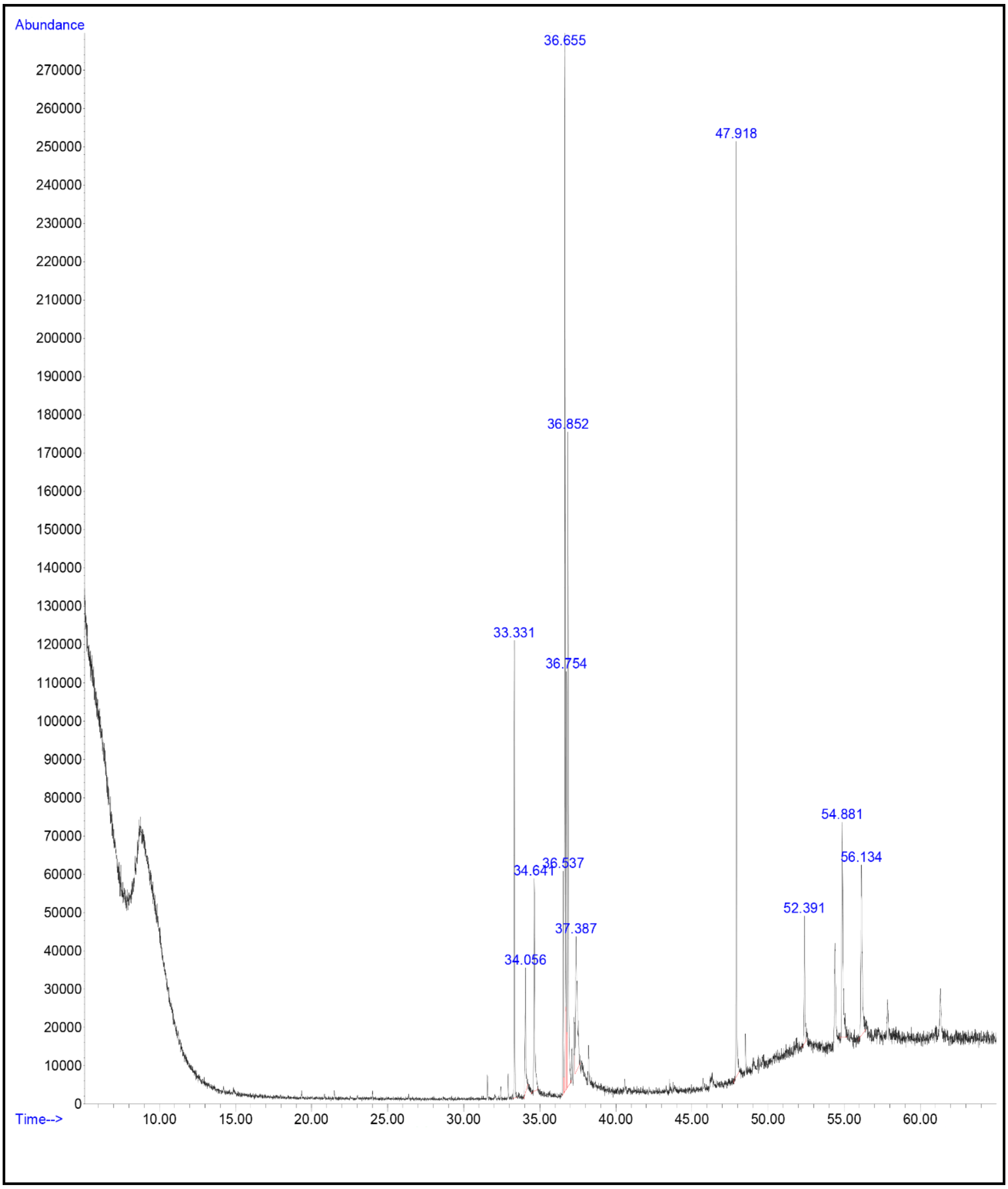

Methyl palmitate

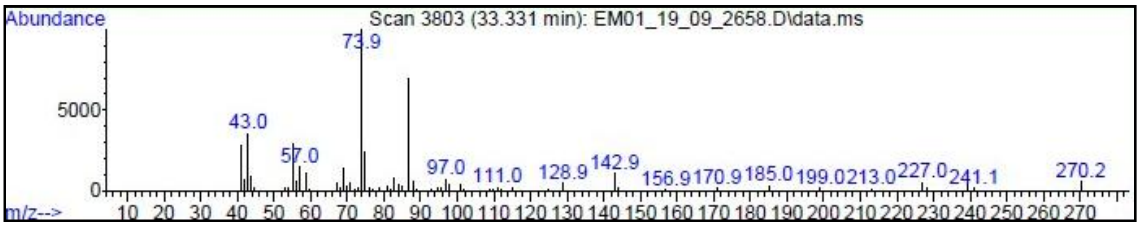

Palmitic acid

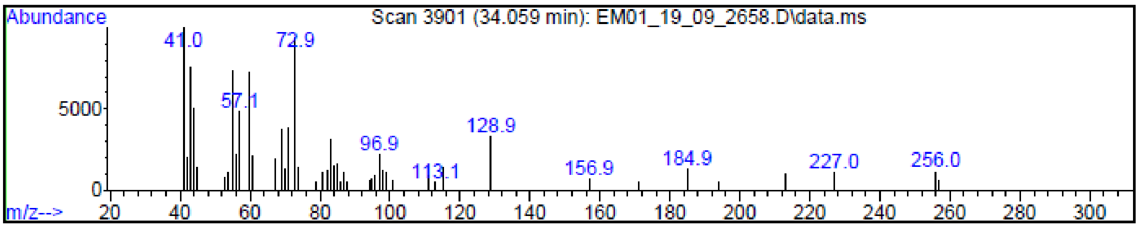

2-azaanthraquinone

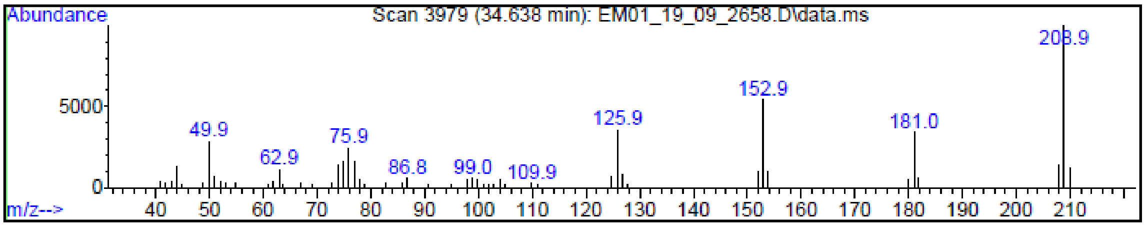

Methyl linoleate

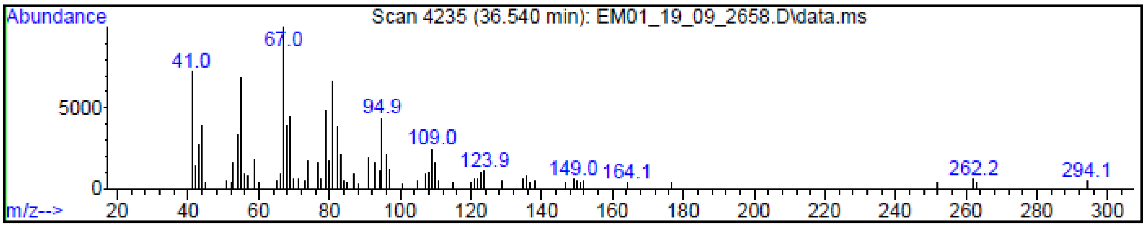

Pentalongin

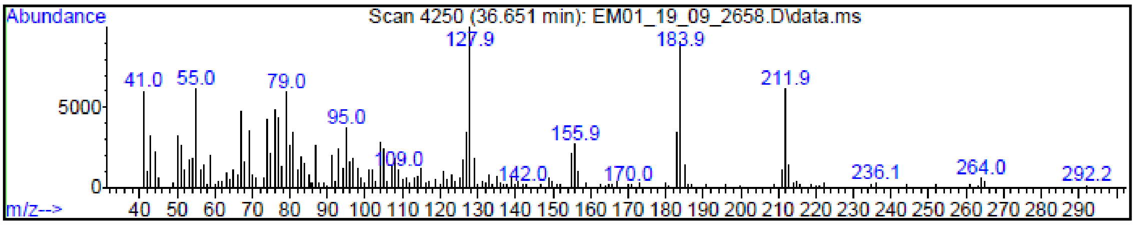

Methyl oleate

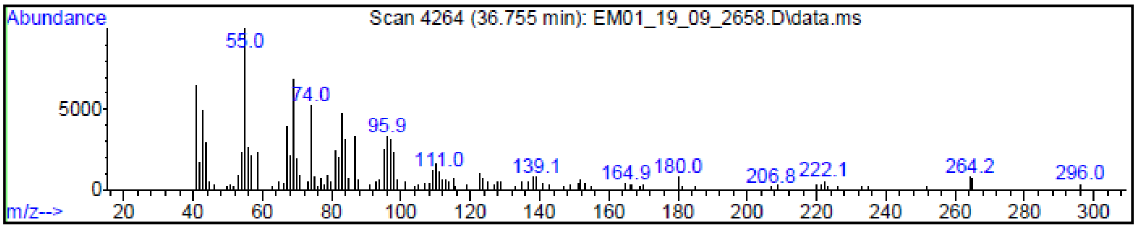

Neophytadiene

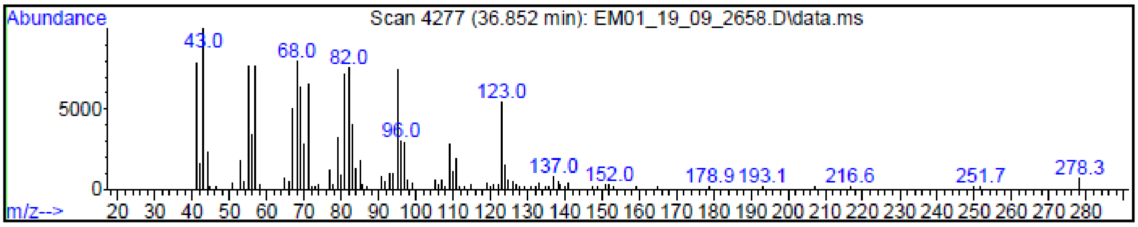

Indeterminate

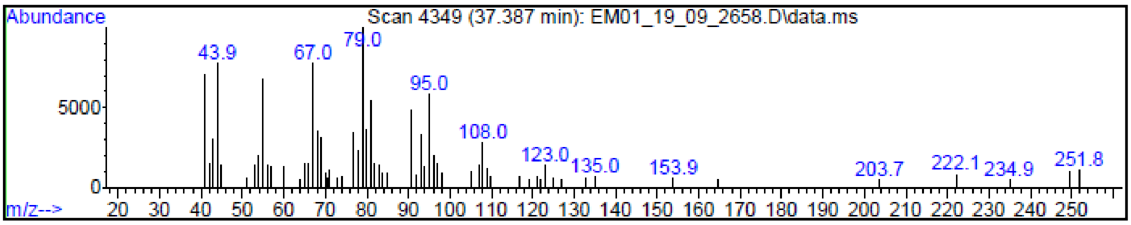

Squalene

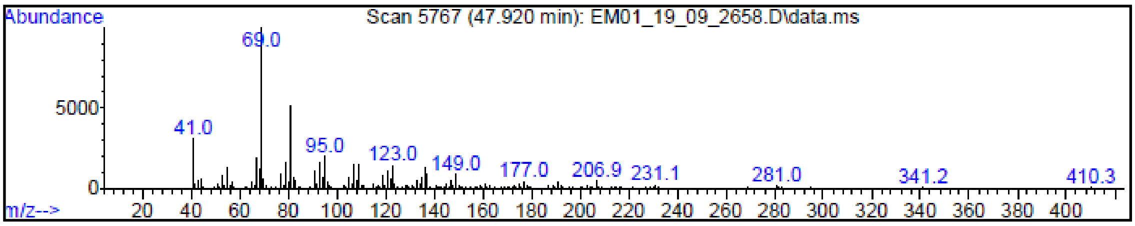

Alpha-tocopherol

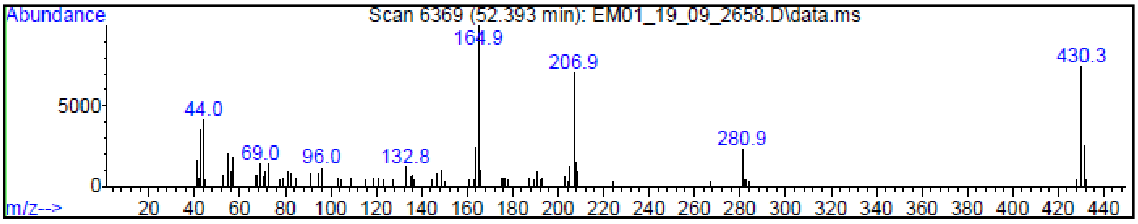

Stigmasterol

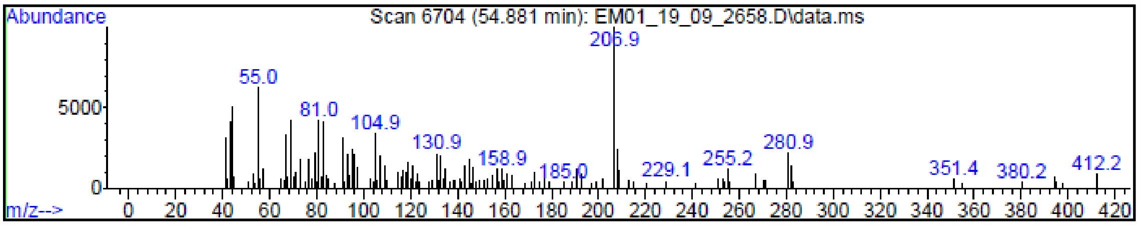

γ- Sitosterol

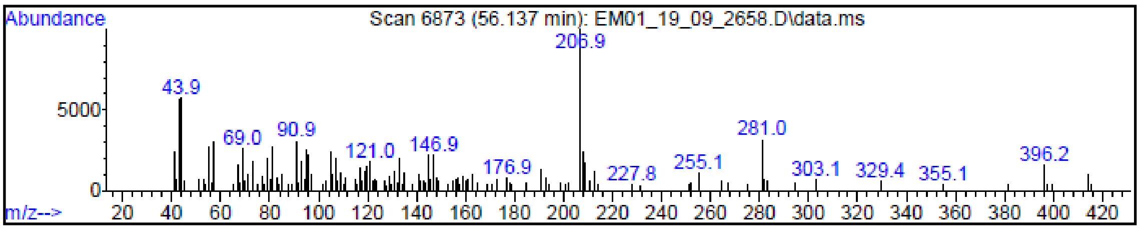

Supplement: Supplementary file 1 [file ao5c05713_si_001.pdf]
